# ﻿Six new species of *Aspidophorodon* Verma, 1967 (Hemiptera, Aphididae, Aphidinae) from China

**DOI:** 10.3897/zookeys.1106.77912

**Published:** 2022-06-16

**Authors:** Ying Xu, Li-Yun Jiang, Jing Chen, Bakhtiyor Rustamovich Kholmatov, Ge-Xia Qiao

**Affiliations:** 1 Key Laboratory of Zoological Systematics and Evolution, Institute of Zoology, Chinese Academy of Sciences, No. 1-5 Beichen West Road, Chaoyang District, Beijing 100101, China Institute of Zoology, Chinese Academy of Sciences Beijing China; 2 Institute of Zoology, Academy of Sciences Republic of Uzbekistan, Bagishamol Str., 232b, Tashkent 100053, Uzbekistan University of Chinese Academy of Sciences Beijing China; 3 College of Life Science, University of Chinese Academy of Sciences, No. 19, Yuquan Road, Shijingshan District, Beijing 100049, China Institute of Zoology, Academy of Sciences Republic of Uzbekistan Tashkent Uzbekistan

**Keywords:** DNA barcode, key, new record, new synonym, NJ tree, *Salix*

## Abstract

The genus *Aspidophorodon* Verma is presented including six new species from China namely *Aspidophorodoncapitatum* Qiao & Xu, **sp. nov.***Aspidophorodonlongicornutum* Qiao & Xu, **sp. nov.***Aspidophorodonreticulatum* Qiao & Xu, **sp. nov.***Aspidophorodonfurcatum* Qiao & Xu, **sp. nov.***Aspidophorodonlongirostre* Qiao & Xu, **sp. nov.** and *Aspidophorodonobtusirostre* Qiao & Xu, **sp. nov.***Aspidophorodoncornuatum* Qiao 2015, is considered as a junior synonym of *Aspidophorodonlongituberculatum* (Zhang Zhong & Zhang 1992), **syn. nov.** Two species *Aspidophorodonharvense* Verma and *Aspidophorodonindicum* (David Rajasingh & Narayanan) are recorded for the first time in China. The genus is mainly distributed in East Asia and is represented by 15 species in the world of which 12 are found in China. Keys to the species of *Aspidophorodon* are given.

## ﻿Introduction

*Aspidophorodon* is a genus of Macrosiphini (Hemiptera: Aphididae: Aphidinae) with two subgenera, the nominate subgenus and subgenus Eoessigia (Favret 2022), mostly feeding on plants of Salicaceae and Rosaceae (Blackman and Eastop 2006). The taxonomy of *Aspidophorodon* was last revised by Chen et al. (2015) with ten species, namely *A.cornuatum* Qiao, *A.harvense* Verma, *A.musaicum* Qiao, *A.obtusum* Qiao, *A.salicis* Miyazaki, A. (Eoessigia) indicum (David, Rajasingh & Narayanan), A. (Eoessigia) longicauda (Richards), A. (Eoessigia) longituberculatum (Zhang, Zhong & Zhang), A. (Eoessigia) sorbi (Chakrabarti & Maity), and A. (Eoessigia) vera Stekolshchikov & Novgorodova.

The genus is distinguished from others as follows: head with three processes on frons; dorsum of body variously decorated with wrinkles, irregular polygonal reticulations, oval or semicircular sculptures, small papillate tubercles; siphunculus long and spoon-shaped, broad at base, slightly swollen distally, without flange. After examining specimens of this genus from China, six new species are here described. In addition, *Aspidophorodoncornuatum* Qiao, 2015 is regarded as a junior synonym of *Aspidophorodonlongituberculatum* (Zhang, Zhong & Zhang, 1992) syn. nov. Two species, *Aspidophorodonharvense* Verma and *Aspidophorodonindicum* (David, Rajasingh & Narayanan) are recorded in China for the first time.

## ﻿Materials and methods

### ﻿Morphological description

Aphid terminology in this paper generally follows that of Chen et al. (2015). The unit of measurement is millimeters (mm). In this paper, the following abbreviations are used:

**Ant. I, II, III, IV, V, VI** antennal segments I, II, III, IV, V, VI;

**Ant. IVb, Vb, VIb** base of segment IV, V or VI, respectively;

**PT** processus terminalis;

**Ant. IIIBD** basal diameter of antennal segment III;

**URS** ultimate rostral segment;

**BW URS** basal width of ultimate rostral segment;

**MW hind tibia** mid-width of hind tibia;

**2HT** second hind tarsal segment;

**SIPH** siphunculus;

**BW SIPH** basal width of siphunculus;

**MW SIPH** mid-width of siphunculus;

**DW SIPH** distal width of siphunculus;

**BW Cauda** basal width of cauda.

### ﻿DNA barcoding

The DNA barcodes of twenty-six samples of *Aspidophorodon* were obtained, including the six new species and seven known species. The aphid samples used in this research and voucher information are listed in Table 1.

**Table 1.** Voucher and GenBank accession numbers for aphid samples used in the molecular analyses.

Species	Voucher number	Collection locality	Host plant	COI
*A.capitatum* Qiao & Xu, sp. nov.	49120	China: Tibet (Bomi)	*Salix* sp.	OK668442
*A.capitatum* Qiao & Xu, sp. nov.	51730	China: Tibet (Bomi)	*Salix* sp.	OK668446
*A.furcatum* Qiao & Xu, sp. nov.	45884	China: Sichuan (Minya Konka)	*Salix* sp.	OK668438
*A.furcatum* Qiao & Xu, sp. nov.	45911	China: Sichuan (Minya Konka)	*Salix* sp.	OK668439
*A.harvense* Verma	45942	China: Sichuan (Ganzi)	*Spiraea* sp.	OK668440
*A.indicum* (David, Rajasingh & Narayanan)	52024	China: Tibet (Jilong)	*Cotoneaster* sp.	OK668434
*A.indicum* (David, Rajasingh & Narayanan)	52044	China: Tibet (Jilong)	*Cotoneaster* sp.	OK668447
*A.longicauda* (Richards)	CNC#HEM114051	Canada	Unknown	KR031700.1*
*A.longicauda* (Richards)	CNC#HEM057620	Canada	Unknown	KR038732.1*
*A.longicauda* (Richards)	CNC#HEM114048	Canada	Unknown	KR038867.1*
*A.longicauda* (Richards)	CNC#HEM057547	Canada	Unknown	KR042463.1*
*A.longicauda* (Richards)	CNC#HEM057563	Canada	Unknown	KR045217.1*
*A.longicornutum* Qiao & Xu, sp. nov.	41008	China: Shaanxi (Ankang)	*Salix* sp.	OK668436
*A.longicornutum* Qiao & Xu, sp. nov.	41027	China: Shaanxi (Ankang)	Unknown	OK668437
*A.longirostre* Qiao & Xu, sp. nov.	15089	China: Sichuan (Baoxing)	*Salix* sp.	OK668432
*A.cornuatum* (Qiao), 2015, syn. nov.	25908	China: Tibet (Yadong)	*Salixcupularis*	KJ374724*
*A.cornuatum* (Qiao), 2015, syn.nov.	51707	China: Tibet (Bomi)	*Salix* sp.	OK668444
*A.longituberculatum* (Zhang, Zhong & Zhang, 1992)	51707 al.	China: Tibet (Bomi)	*Salix* sp.	OK668445
*A.musaicum* Qiao	17257	China: Sichuan (Meigu)	Unknown	KJ374722*
*A.obtusum* Qiao	22562	China: Sichuan (Minya Konka)	*Salix* sp.	KJ374723*
*A.obtusum* Qiao	47777	China: Sichuan (Minya Konka)	*Cotoneaster* sp.	OK668441
*A.reticulatum* Qiao & Xu, sp. nov.	37265	China: Tibet (Cuona)	*Salixcupularis*	OK668435
*A.salicis* Miyazaki	15038	China: Sichuan (Baoxing)	*Salix* sp.	KT221040*
*A.salicis* Miyazaki	49999	China: Beijing	*Salix* sp.	OK668443
*A.salicis* Miyazaki	23167	China: Sichuan (Leshan)	*Salix* sp.	KT221041*
*A.obtusirostre* Qiao & Xu, sp. nov.	35918	China: Beijing (Mt. Dongling)	Unknown	OK668433

*Sequences downloaded from GenBank.

The methods of extracting DNA and PCR thermal regime followed those of Chen et al. (2015). Sequences were assembled by SeqMan II (DNAStar, Inc., Madison, WI, USA) with inspection and manual editing, and then were examined using BLAST to confirm the sequences were highly similar to aphid species. All sequences were deposited in GenBank (Table 1). Multiple alignments were performed with ClustalW (Thompson et al. 1994) and then verified manually. Pairwise genetic distances and Neighbor-joining (NJ) tree for COІ gene were estimated using MEGA7 (Kumar et al. 2016) under Kimura’s two-parameter (K2P) model (Kimura 1980). Bootstrap analyses were performed with 1000 replicates.

### ﻿Specimen depositories

The holotypes and some paratypes of six new species and other specimens examined are deposited in the National Animal Collection Resource Center, Institute of Zoology, Chinese Academy of Sciences, Beijing, China (unmarked in the text). The other paratypes of new species are deposited in the Natural History Museum, London, UK (**NHMUK**, marked in the text).

## ﻿Taxonomy

### ﻿ Aspidophorodon

Taxon classificationAnimaliaHemipteraAphididae

Verma, 1967

FED1D191-7309-5C9D-9B37-74598C94DDD1

Aspidophorodon Verma 1967: 507. Type species: AspidophorodonharvenseVerma 1967; by original designation. Miyazaki 1971: 183; Eastop and Hille Ris Lambers 1976: 95; Remaudière and Remaudière 1997: 73; Zhang et al. 1999: 349; Blackman and Eastop 2006: 1098; Stekolshchikov and Novgorodova 2010: 44; Nieto Nafría et al. 2011: 145; Chen et al. 2015: 557.
Indotuberoaphis Chakrabarti & Maity 1984: 198. Type species: Indotuberoaphissorbi Chakrabarti & Maity 1984; by original designation.
Margituberculatus Zhang, Zhong & Zhang 1992: 381. Type species: Margituberculatuslongituberculatum Zhang, Zhong & Zhang 1992; by original designation.
Raychaudhuriella Chakrabarti 1978: 355. Type species: RaychaudhuriellamyzaphoidesChakrabarti 1978: 357; by original designation.

#### Generic diagnosis.

Head with three processes on frons; median frontal tubercle in apterae distinctly protuberant, hemispherical, rectangular, or forked, sometimes with a depression at the middle; antennal tubercles undeveloped, but each with a cylindrical, finger-shaped, or long horn-shaped process at inner apex, the process higher or lower than median frontal tubercle in apterae. Body dorsum with various markings in apterous viviparous females: wrinkles, irregular polygonal reticulations, oval or semicircular sculptures, or small papillate tubercles. Antennae 4- or 5-segmented (rarely 6-segmented) in apterae, 5- or 6-segmented in alatae. Ant. I usually rounded or projected to short cylindrical at inner apex. Secondary rhinaria present on antennal segments III–V in alatae. SIPH spoon-shaped, broad at base, thin at the middle, slightly swollen distally, obliquely truncated at tip, without flange. Cauda tongue-shaped or elongate conical, slightly constricted near the middle, sometimes with a constriction at base, with 4–5 setae, rarely 6–11.

#### Distribution.

Canada, China, India, Japan, Russia (Sakhalin, the Altai Republic, and the Kuril Islands), and Kashmir region.

#### Host plants.

Rosaceae (*Cotoneaster*, *Potentilla*, *Sorbus*, *Spiraea*), and Salicaceae (*Salix*), rarely on Polygonaceae (*Polygonum*).

#### Comments.

The genus Aspidophorodon includes two subgenera, the nominate subgenus and subgenus Eoessigia. The most important difference between the two subgenera is the presence of at least one spinal process on abdominal tergite VIII in Aspidophorodon (Eoessigia), whereas no such spinal process is found on members of the nominate subgenus. See Chen et al. (2015) for a key to subgenera.

### Subgenus ﻿ Aspidophorodon

Taxon classificationAnimaliaHemipteraAphididae

Verma, 1967

372DC4A2-7F0A-5C57-AA64-19F29A59FAAB

Aspidophorodon Verma 1967: 507. Type species: AspidophorodonharvenseVerma 1967; by original designation.
Aspidophorodon (Aspidophorodon) Verma: Remaudière and Remaudière 1997: 73; Stekolshchikov and Novgorodova 2010: 44; Nieto Nafría et al. 2011: 145; Chen et al. 2015: 557.

#### Comments.

Spinal processes on body dorsum absent, and marginal processes present or absent on thoracic nota and abdominal tergites I–IV in apterae. Median frontal tubercle protuberant, hemispherical, rectangular, sometimes with a depression at the middle in apterae. Antennae 4- or 5-segmented in apterae. Cauda tongue-shaped with 4 or 5 setae, sometimes with 6 setae.

The nominate subgenus contains seven species, including three new species. *Aspidophorodonharvense* Verma is first recorded in China. This subgenus is mainly distributed in eastern Asia.

### ﻿Key to the species of Aspidophorodon (Aspidophorodon) (based on apterous viviparous females)

**Table:** 

1	Marginal processes on thoracic nota and abdominal tergites absent	**2**
–	Marginal processes on thoracic nota and abdominal tergites present	**5**
2	Head with three inconspicuous processes on frons, median frontal tubercle moderately protuberant; antennal tubercles each with an inconspicuous process at inner apex; Ant. I rounded at inner apex; body dorsum scabrous with many small papillate tubercles; body dorsal setae extremely thick long and capitate, on swollen setal tubercles	***A.capitatum* Qiao & Xu, sp. nov.**
–	Head with three developed processes on frons, median frontal tubercle rectangular or hemispherical; antennal tubercles each with a short cylindrical or horn-shaped process at inner apex; Ant. I projected at inner apex; body dorsum with reticulations, without small papillate tubercles; body dorsal setae short and thin, blunt or pointed at apices, on normal setal tubercles	**3**
3	Median frontal tubercle with a depression at the middle; process at inner apex of antennal tubercle as high as median frontal tubercle	***A.salicis* Miyazaki**
–	Median frontal tubercle without a depression at the middle; process at inner apex of antennal tubercle much higher than median frontal tubercle	**4**
4	Antennal tubercles each with a long horn-shaped process at inner apex, 2.40–2.50 × as long as its basal width; body dorsum with distinctly oval and polygonal reticulations	***A.harvense* Verma**
–	Antennal tubercles each with a cylindrical process at inner apex, 1.82–2.04 × as long as its basal width; body dorsum with reticulations consisting of small triangles arranged in polygons	***A.reticulatum* Qiao & Xu, sp. nov.**
5	Antennal tubercles each with a short cylindrical process at inner apex, the process lower than median frontal tubercle; marginal processes on meso- and metanotum and abdominal tergites I–IV distinctly long and horn-shaped	***A.longicornutum* Qiao & Xu, sp. nov.**
–	Antennal tubercles each with a cylindrical process at inner apex, the process higher than median frontal tubercle; marginal processes on thoracic nota and abdominal tergites I–IV cylindrical	**6**
6	Abdominal tergites with irregular polygonal mosaic-like markings; marginal processes on thoracic nota and abdominal tergites I–IV long and tapered	***A.musaicum* Qiao**
–	Abdominal tergites with reticulations formed by small irregular oval markings; marginal processes on thoracic nota and abdominal tergites I–IV cylindrical, with obtuse apices	***A.obtusum* Qiao**

### ﻿ Aspidophorodon capitatum

Taxon classificationAnimaliaHemipteraAphididae

Qiao & Xu sp. nov.

34A1EF54-6C8B-5D19-825F-95AF84266B2C

http://zoobank.org/101581BE-83AD-4F7B-9C28-BEB8F727838F

**Figure 1.**
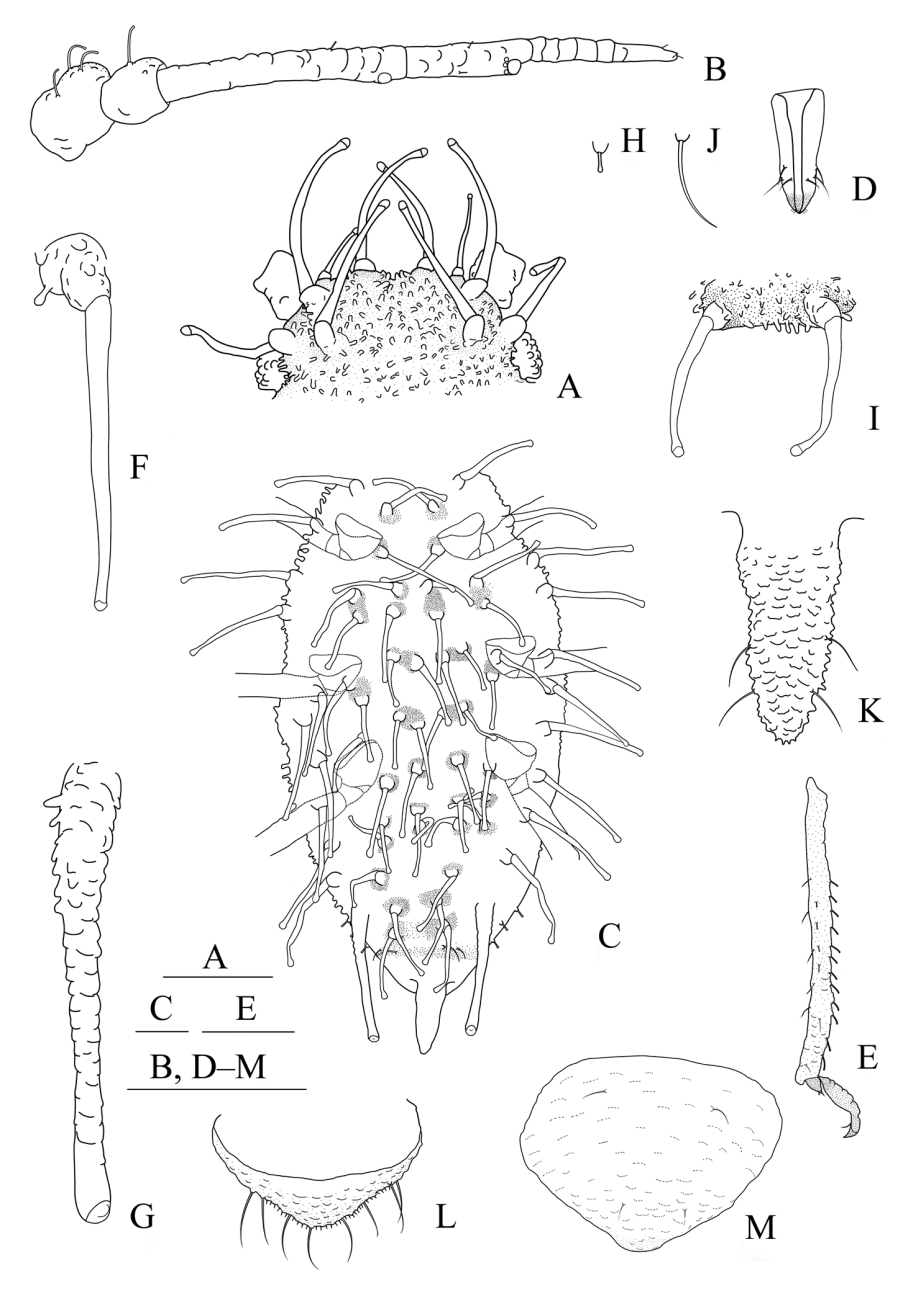
*Aspidophorodoncapitatum* Qiao & Xu, sp. nov. Apterous viviparous female **A** dorsal view of head **B** antenna **C** dorsal view of thorax and abdomen **D** ultimate rostral segment **E** hind tibia and tarsi **F** marginal seta of abdominal tergite I **G** siphunculus **H** marginal seta of abdominal tergite V **I** spinal setae of abdominal tergite VII **J** spinal seta of abdominal tergite VIII **K** cauda **L** anal plate **M** genital plate. Scale bars: 0.10 mm.

**Figure 2.**
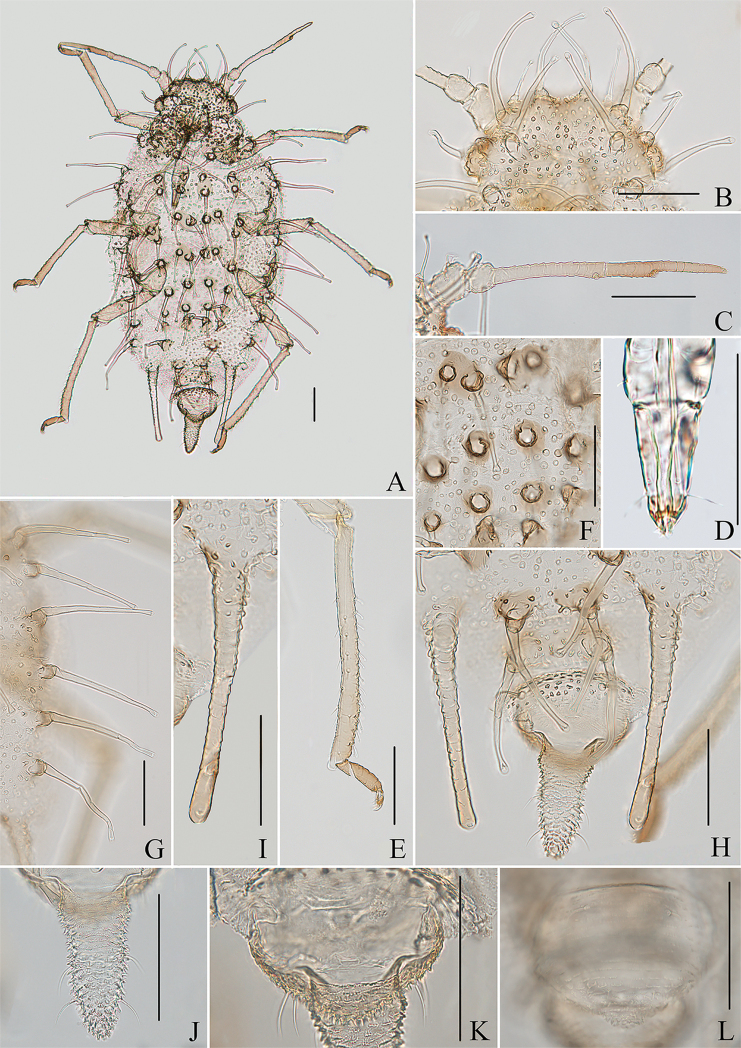
*Aspidophorodoncapitatum* Qiao & Xu, sp. nov. Apterous viviparous female **A** dorsal view of body **B** dorsal view of head **C** antenna **D** ultimate rostral segment **E** hind tibia and tarsi **F** papillated tubercles at seta-basal of abdominal tergites **G** marginal setae of metanotum and abdominal tergites I–IV **H** dorsal view of abdominal tergites VI–VIII **I** siphunculus **J** cauda **K** anal plate **L** genital plate. Scale bars: 0.10 mm.

Figs 1 , 2 , 21A , Table 2

#### Specimens examined.

***Holotype***: apterous viviparous female, China, Tibet (Bomi County, 30.15°N, 94.99°E, altitude 2160 m), 01.IX.2020, No. 49120-1-1-2, on *Salix* sp., coll. Y. Xu. ***Paratypes***: one apterous viviparous female (slide), No. 49120-1-1-1, one apterous viviparous female (COI: OK668442), and four fourth instar apterous nymphs, with the same collection data as holotype; one apterous viviparous female, 26.VI.2021, No. 51696-1-1, on *Salix* sp., coll. Y. Xu; one apterous viviparous female (slide) and one apterous viviparous female (COI: OK668446), 29.VI.2021, No. 51730-1-2, on *Salix* sp., coll. Y. Xu; one apterous viviparous female, No. 49120-1-2 (NHMUK), with the same collection data as holotype.

#### Diagnosis.

Dorsum of body densely covered with small papillate tubercles; median frontal tubercle moderately protuberant, with a shallow depression at the middle; antennal tubercles each with an inconspicuous process at inner apex lower than median frontal tubercle; dorsal setae of body distinctly long, thick, capitate, on swollen setal tubercles.

#### Description.

Apterous viviparous females: body elongated oval (Fig. 2A), yellowish white in life (Fig. 21A).

**Mounted specimens.** Body pale; head, compound eyes, Ant. IV, distal part of rostrum, legs, setal tubercles, distal part of SIPH, cauda and anal plate pale brown; tarsi brown. Thoracic nota and abdominal tergites I–IV each with one pair of spinal and one pair of pleural sclerites, tergites V–VII each with one pair of spinal sclerites, those sclerites pale brown in color; tergite VIII with a pale brown band (Figs 1C, 2A); other parts pale in color. See Table 2 for general measurements.

**Table 2.** Morphometric data about species of the nominate subgenus (in mm).

Parts		*A.capitatum* Qiao & Xu, sp. nov.		*A.harvense* Verma		*A.longicornutum* Qiao & Xu, sp. nov.	*A.reticulatum* Qiao & Xu, sp. nov.
		Apterous viviparous female (n = 4)	4^th^ apterous nymph (n = 4)	Apterous viviparous female (n = 2)	Alate viviparous female (n = 1)	Apterous viviparous female (n = 7)	Apterous viviparous female (n = 2)
Length (mm)	Body length	1.001–1.104	0.817–0.894	2.147–2.238	1.884	0.899–1.079	1.743–1.753
	Body width	0.448–0.540	0.408–0.497	0.966–1.091	0.741	0.382–0.467	0.908–0.940
	Antennae	0.362–0.374	0.324–0.338	0.806–0.828	1.034	0.330–0.422	0.569–0.537
	Ant. I	0.051–0.053	0.048–0.051	0.086–0.088	0.080	0.046–0.055	0.062–0.067
	Ant. II	0.036–0.038	0.032–0.038	0.062–0.070	0.067	0.033–0.039	0.044–0.048
	Ant. III	0.130–0.144	0.102–0.119	0.293–0.324	0.326	0.125–0.178	0.170–0.181
	Ant. IV	/	/	0.145–0.161	0.152	/	0.101–0.103
	Ant. IVb	0.060–0.067	0.057–0.066	/	/	0.061–0.081	/
	Ant. V	/	/	/	0.188	/	/
	Ant. Vb	/	/	0.107	/	/	0.082–0.084
	Ant. VIb	/	/	/	0.113	/	/
	PT	0.081–0.084	0.071–0.083	0.094–0.097	0.108	0.064–0.075	0.097–0.104
	URS	0.076–0.079	0.074–0.083	0.082–0.092	0.082	0.084–0.095	0.124–0.128
	Hind femur	0.164–0.170	0.123–0.145	0.394–0.410	0.48	0.144–0.178	0.288–0.290
	Hind tibia	0.327–0.334	0.272–0.299	0.724–0.765	0.974	0.255–0.315	0.540–0.560
	2HT	0.058–0.061	0.059–0.065	0.114–0.119	0.122	0.056–0.067	0.080–0.082
	SIPH	0.226–0.257	0.175–0.203	0.362–0.381	0.269	0.244–0.331	0.256–0.263
	BW SIPH	0.029–0.031	0.032–0.044	0.064–0.069	0.036	0.036–0.049	0.047–0.049
	MW SIPH	0.014–0.015	0.014–0.016	0.042–0.049	0.026	0.012–0.014	0.028–0.030
	DW SIPH	0.019–0.020	0.017–0.018	0.031–0.035	0.035	0.016–0.018	0.028–0.032
	Cauda	0.126–0.136	/	0.190	0.134	0.086–0.112	0.149–0.162
	BW Cauda	0.071–0.078	/	0.110–0.117	0.090	0.045–0.057	0.097–0.103
	MW Cauda	/	/	0.082–0.086	0.046	0.032–0.037	0.062–0.063
	Ant. IIIBD	0.018–0.020	0.021	0.033–0.037	0.033	0.017–0.022	0.025–0.026
	Widest width of hind femur	0.045–0.048	0.044–0.050	0.076–0.078	0.067	0.036–0.045	0.062–0.068
	MW hind tibia	0.022–0.024	0.028–0.032	0.039–0.041	0.034	0.019–0.021	0.036
	Cephalic setae	0.124–0.132	0.075–0.084	0.020–0.023	0.023	0.022–0.033	0.024
	Dorsal setae of head	/	/	0.006–0.008	0.013	/	0.023–0.024
	Dorsal setae of head between antenna	0.157–0.161	0.105–0.119	/	/	0.021–0.030	/
	Dorsal setae of head between compound eyes	0.139–0.175	0.076–0.111	/	/	0.029–0.034	/
Length (mm)	Marginal setae on Tergite I	0.193–0.198	0.118–0.129	0.006–0.010	0.014	0.026–0.033	0.014–0.016
	Spinal setae on Tergite VIII	0.018–0.021	0.013–0.019	0.019–0.025	0.031	0.027–0.035	0.025–0.028
	Setae on Ant. III	0.008–0.010	0.005–0.007	0.006–0.007	0.011	/	0.010–0.012
	Setae on hind tibia	0.017–0.019	0.028–0.036	0.016–0.017	0.021	0.014–0.02	0.054–0.056
	Processes on antennal tubercle	/	/	0.110–0.120	0.019	0.015–0.026	0.051–0.056
	Marginal process on mesonotum	/	/	/	/	0.268–0.325	/
	Marginal process on metanotum	/	/	/	/	0.244–0.286	/
	Marginal process on Tergite I	/	/	/	/	0.211–0.26	/
	Marginal process on Tergite II	/	/	/	/	0.224–0.265	/
	Marginal process on Tergite III	/	/	/	/	0.233–0.288	/
	Marginal process on Tergite IV	/	/	/	/	0.234–0.309	/
Ratio (times)	Body length / Body width	2.00–2.23	1.80–2.08	2.05–2.22	2.54	2.23–2.81	1.85–1.93
	Whole antennae / Body	0.33–0.37	0.37–0.41	0.36–0.39	0.55	0.34–0.40	0.33
	Hind femur / Ant. III	1.18–1.27	1.10–1.42	1.22–1.40	1.47	1.00–1.21	1.60–1.69
	Hind tibia / Body	0.30–0.33	0.30–0.37	0.32–0.36	0.52	0.28–0.30	0.31–0.32
	Ant. I / Ant. III	0.35–0.41	0.43–0.50	0.27–0.29	0.25	0.29–0.38	0.37
	Ant. II / Ant. III	0.26–0.29	0.27–0.36	0.21–0.22	0.21	0.21–0.27	0.24–0.28
	Ant. IV / Ant. III	/	/	0.45–0.55	0.47	/	0.56–0.61
	Ant. V/ Ant. III	/	/	/	0.58	/	/
	Ant. IVb, Vb or VIb / Ant. III	0.42–0.52	0.52–0.59	0.33–0.37	0.35	0.43–0.49	0.46–0.48
	PT / Ant. III	0.56–0.65	0.62–0.81	0.29–0.33	0.33	0.40–0.52	0.54–0.61
	PT / Ant. IVb, Vb or VIb	1.25–1.38	1.08–1.43	0.88–0.91	0.96	0.88–1.09	1.16–1.27
	URS / BW URS	2.03–2.05	1.95–2.36	1.39–1.63	1.55	2.37–2.85	2.70–2.72
	URS / 2HT	1.28–1.32	1.20–1.41	0.69–0.81	0.67	1.31–1.59	1.51–1.61
	SIPH / Body	0.22–0.26	0.21–0.24	0.16–0.17	0.14	0.27–0.31	0.15
	SIPH / Cauda	1.66–1.88	/	1.91–2.01	2.01	2.64–3.07	1.58–1.77
	SIPH / BW SIPH	7.29–8.86	3.98–5.88	5.22–5.66	7.47	6.10–7.15	5.22–5.66
	SIPH / MW SIPH	16.14–17.13	11.67–12.69	7.78–8.62	10.35	20.00–24.31	8.74–9.14
	SIPH / DW SIPH	11.85–12.85	10.29–11.29	10.34–12.29	7.69	13.56–19.47	8.23–9.14
	Cauda / BW Cauda	1.75–1.92	/	1.62–1.73	1.49	1.78–2.24	1.53–1.57
	Cephalic setae / Ant. IIIBD	6.30–6.89	3.75–4.00	0.61–0.62	0.7	1.22–1.71	0.92–0.98
	Marginal setae on Tergite I / Ant. III BD	9.76–10.78	5.62–6.40	0.18–0.27	0.42	1.24–1.29	0.54–0.66
Ratio (times)	Spinal setae on Tergite VIII / Ant. III BD	2.50–3.11	1.62–2.00	0.51–0.76	0.94	1.50–1.82	0.96–1.14
	Setae on Ant. III / Ant. IIIBD	0.40–0.50	0.24–0.33	0.18–0.19	0.33	/	0.43–0.46
	Setae on hind tibia / MW hind tibia	0.77–0.79	1.00–1.29	0.41–0.42	0.62	0.74–0.95	0.60–0.67
	Length of processes on antennal tubercle / its basal width	/	/	2.40–2.50	0.61	0.89–1.73	1.82–2.04
	Length of marginal process on mesonotum / its basal width	/	/	/	/	7.05–10.48	/
	Length of marginal process on mesonotum / SIPH	/	/	/	/	0.86–1.11	/
	Length of marginal process on metanotum / its basal width	/	/	/	/	6.95–9.53	/
	Length of marginal process on metanotum / SIPH	/	/	/	/	0.81–1.00	/
	Length of marginal process on Tergite I / its basal width	/	/	/	/	5.90–6.89	/
	Length of marginal process on Tergite I / SIPH	/	/	/	/	0.74–0.88	/
	Length of marginal process on Tergite II / its basal width	/	/	/	/	5.88–8.55	/
	Length of marginal process on Tergite II / SIPH	/	/	/	/	0.79–0.96	/
	Length of marginal process on Tergite III / its basal width	/	/	/	/	6.68–9.32	/
	Length of marginal process on Tergite III / SIPH	/	/	/	/	0.87–1.02	/
	Length of marginal process on Tergite IV / its basal width	/	/	/	/	6.58–8.36	/
	Length of marginal process on Tergite IV / SIPH	/	/	/	/	0.90–0.96	/

***Head*.** Ocular tubercles small. Dorsum of head densely covered with small papillate tubercles (Figs 1A, 2B), venter with wrinkles and sparse small papillate tubercles. Median frontal tubercle moderately protuberant, with a shallow depression at the middle (Figs 1A, 2B), with one pair of long capitate setae on venter. Antennal tubercles undeveloped, each with an inconspicuous process at inner apex, lower than median frontal tubercle, each process with a long capitate seta at apex (Figs 1A, 2B). Dorsal setae of head extremely long, thick, capitate, on swollen setal tubercles which are covered with sparsely small papillae. Head with one pair of cephalic setae, one pair of dorsal setae between antennae, and two pairs of dorsal setae between compound eyes arranged transversely (Figs 1A, 2B). Antennae 4-segmented, Ant. I–II with wrinkles, Ant. III–IV slightly imbricated; Ant. I rounded at inner apex (Figs 1B, 2C). Antennal setae long, thick and capitate on Ant. I–II, short and blunt on Ant. III–IV; Ant. I–IV with 3, 1-2, 1-2, 2 (base) +0-1 (PT) setae, respectively; apex of PT with two or three setae. Primary rhinaria not ciliated. Rostrum reaching mid-coxae, URS wedge-shaped (Figs 1D, 2D), with three pairs of primary setae, without accessory setae.

***Thorax*.** Pronotum densely covered with small papillate tubercles, meso- and metanotum with small papillate tubercles, distinctly developed on marginal area. Dorsal setae of thorax extremely long, thick, capitate, on swollen setal tubercles which are covered with sparsely small papillae; pronotum with two pairs of spinal setae, arranged anteriorly and posteriorly, one pair of pleural and one pair of marginal setae; meso-, and metanotum with two pairs of spinal, pleural, and marginal setae, respectively (Figs 1C, 2A). Legs normal. Distal parts of femora and tibiae slightly imbricated. Setae on 2/3 distal part of femora and hind tibiae short, blunt ventrally and capitate dorsally (Figs 1E, 2E). First tarsal chaetotaxy: 3, 2, 2. Second tarsal segments with imbrications.

**Figure 3.**
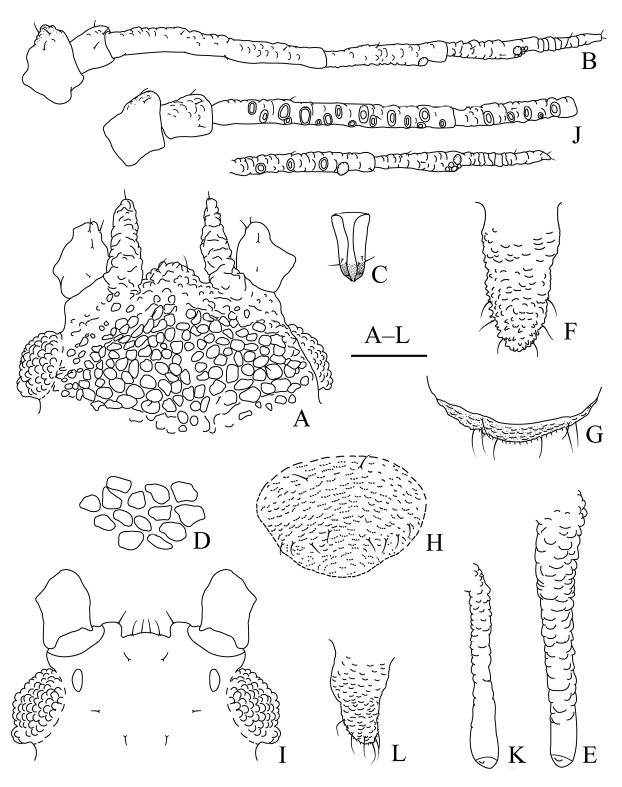
*Aspidophorodonharvense* Verma. Apterous viviparous female **A** dorsal view of head **B** antenna **C** ultimate rostral segment **D** irregular polygonal markings on abdominal tergites **E** siphunculus **F** cauda **G** anal plate **H** genital plate. Alate viviparous female **I** dorsal view of head **J** antenna **K** siphunculus **L** cauda. Scale bar: 0.10 mm.

***Abdomen*.** Abdominal tergites with small papillate tubercles (Fig. 2F), distinctly developed on marginal area. Venter of abdominal tergites III–VIII with fine spinules arranged in rows. Dorsal setae of abdomen extremely long, thick, capitate, on swollen setal tubercles which are covered with small papillae (Figs 1C, 1F, 1I, 2G); the marginal setae of tergites V–VII short, thin, and capitate (Fig. 1H), the setae on tergite VIII long and pointed (Fig. 1J); ventral setae short and pointed. Abdominal tergite I with two pairs of spinal, one pair of pleural and one pair of marginal setae, tergites II–IV each with one pair of spinal, pleural and marginal setae, tergite V with one pair of pleural and one pair of marginal setae, tergites VI–VIII with one pair of spinal and one pair of marginal setae (Figs 1C, 2A). Length of marginal setae on abdominal tergites I–IV, marginal setae on abdominal tergites V–VII, spinal and marginal setae on abdominal tergite VIII 9.65–10.78, 0.60–0.67, 2.50–3.11, 0.90–1.17 × as long as Ant. IIIBD, respectively. Spiracles reniform and open. SIPH long, spoon-shaped, broad at base, thin at the middle, slightly swollen distally; basal part with small papillate tubercles, other parts with imbrications, obliquely truncated at tip, without flange (Figs 1G, 2I). Cauda elongate, conical, slightly constricted at the middle, with spinulose imbrications (Figs 1K, 2J) and four setae. Anal plate semicircular, hind margin slightly protruding backwards, spinulose (Figs 1L, 3K), with 6–9 setae. Genital plate broadly round with sparse spinules in transverse rows, hind margin slightly protruding backwards (Figs 1M, 2L); with two anterior setae and two setae along the posterior margin.

**Fourth instar apterous nymph.** As in apterous viviparous females, except setae on legs long and pointed, and with a row of short and blunt setae dorsally on middle of hind tibiae.

#### Etymology.

The species is named for its extremely long, thick and capitate setae, *capitatum* being the neuter form of the adjective.

#### Taxonomic notes.

The new species resembles *A.harvense* Verma, but differs from it as follows: dorsum of body scabrous, with densely distributed, small, papillate tubercles (the latter: dorsum of body with irregular polygonal markings); median frontal tubercle moderately protuberant, with a shallow depression at middle, antennal tubercles each with an inconspicuous process at inner apex, lower than median frontal tubercle (the latter: median frontal tubercle hemispherical, without a depression at middle, antennal tubercles each with a long horn-shaped process at inner apex, higher than median frontal tubercle); dorsal setae of body extremely long, thick, and capitate, with swollen bases (dorsal setae of body short, thin, and blunt, with normal bases).

#### Host plant.

*Salix* sp.

#### Distribution.

China (Tibet).

#### Biology.

The species dispersedly feeds on the undersides of leaves (Fig. 21A).

### ﻿ Aspidophorodon harvense

Taxon classificationAnimaliaHemipteraAphididae

Verma, 1967

5CFFF1BA-D732-57D0-9465-688CBACF4F73

**Figure 4.**
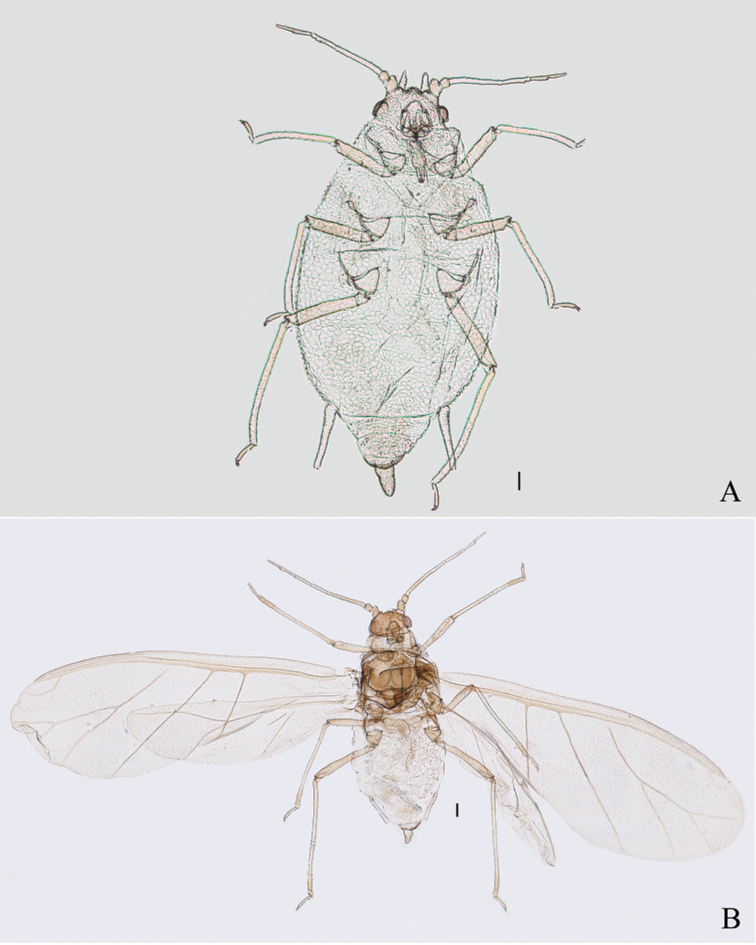
*Aspidophorodonharvense* Verma **A** dorsal view of apterous viviparous female **B** dorsal view of alate viviparous female. Scale bar: 0.10 mm.

**Figure 5.**
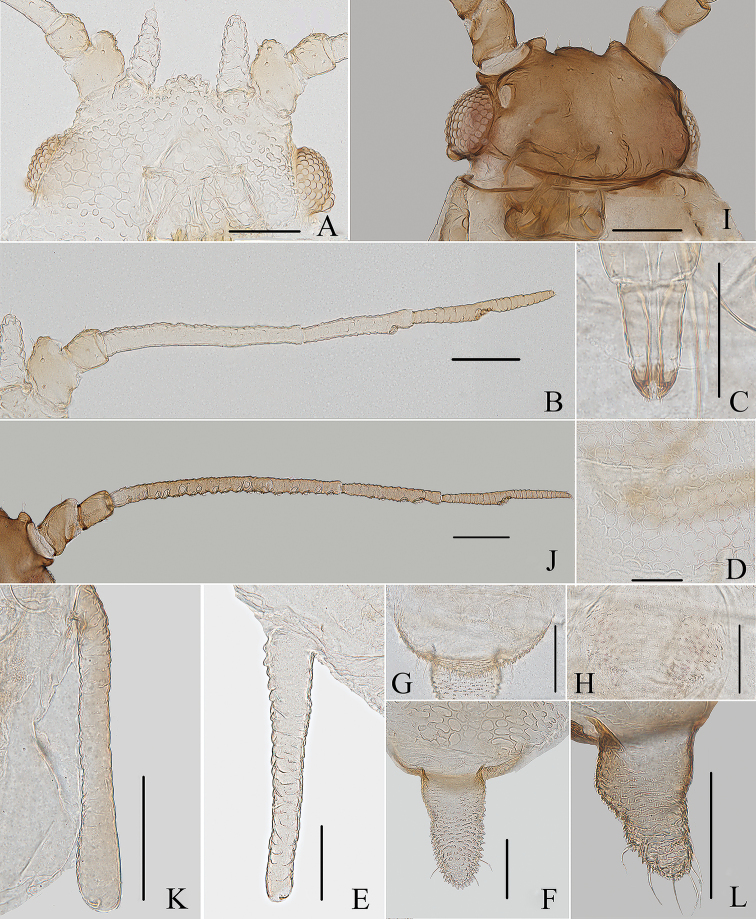
*Aspidophorodonharvense* Verma. Apterous viviparous female **A** dorsal view of head **B** antenna **C** ultimate rostral segment **D** irregular polygonal markings on abdominal tergites **E** siphunculus **F** cauda **G** anal plate **H** genital plate. Alate viviparous female **I** dorsal view of head **J** antenna **K** siphunculus **L** cauda. Scale bars: 0.10 mm.

Figs 3 , 4 , 5 , 21F , Table 2

Aspidophorodon harvense Verma 1967: 507; Eastop and Hille Ris Lambers 1976: 96; Blackman and Eastop 1994: 569.
Aspidophorodon (Aspidophorodon) harvense Verma: Remaudière and Remaudière 1997: 73; Stekolshchikov and Novgorodova 2010: 44; Chen et al. 2015: 558.

#### Specimens examined.

Two apterous viviparous females (slides) and one apterous viviparous female (COI: OK668440), China: Sichuan (Ganzi City, Minya Konka, 29.90°N, 102.03°E, altitude 4031 m), 30.VII.2019, No. 45939-1-1, No. 45942-1-1-2, on *Spiraea* sp., coll. J.F. Ji; one alate viviparous female, No. 45942-1-1-1, with the same collection data as apterous viviparous females.

#### Comment.

*Aspidophoron* being neuter, the adjectival specific epithet is also neuter, so *harvensis* is revised as *harvense*.

#### Host plant.

*Spiraea* sp. (Rosaceae) (Fig. 21F), however, this species was collected from *Salix* sp. in Kashmir in May (Verma 1967).

#### Distribution.

China (Sichuan), Kashmir.

#### Biology.

The species mostly colonizes along veins on the undersides of leaves (Verma 1967).

### ﻿ Aspidophorodon longicornutum

Taxon classificationAnimaliaHemipteraAphididae

Qiao & Xu sp. nov.

EA55AC71-FA51-58C9-92B7-668DD68867C3

http://zoobank.org/C13CA9E8-905F-44D7-B240-63268FA0691C

**Figure 6.**
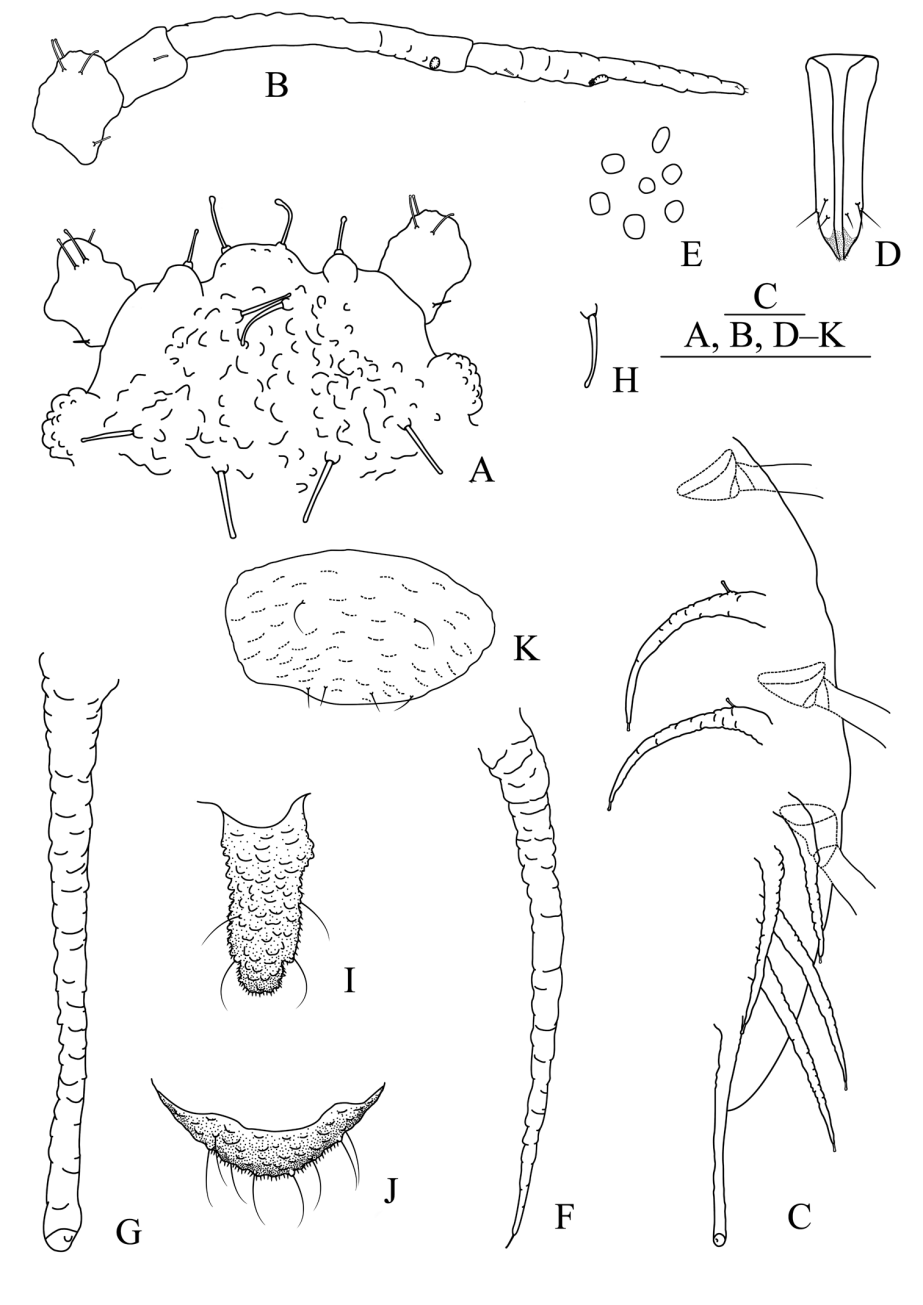
*Aspidophorodonlongicornutum* Qiao & Xu sp. nov. Apterous viviparous female **A** dorsal view of head **B** antenna **C** marginal processes of thoracic nota and abdominal tergites I–IV **D** ultimate rostral segment **E** oval sculptures of abdominal tergites **F** marginal process of abdominal tergite IV **G** siphunculus **H** spinal seta of abdominal tergite VIII **I** cauda **J** anal plate **K** genital plate. Scale bars: 0.10 mm.

**Figure 7.**
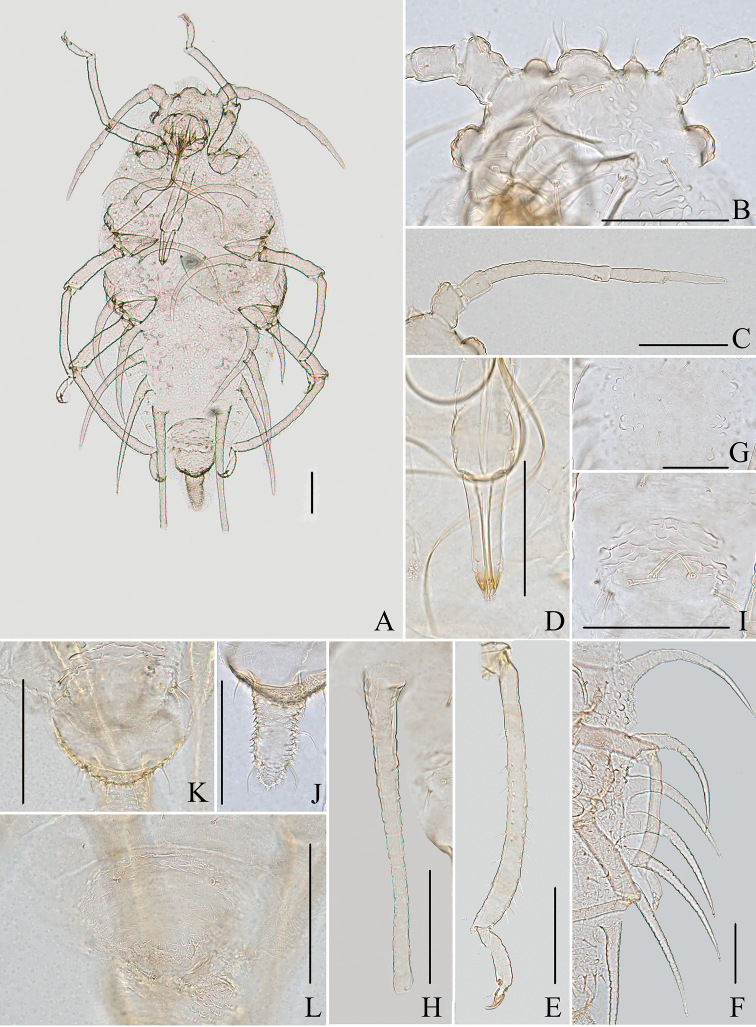
*Aspidophorodonlongicornutum* Qiao & Xu, sp. nov. Apterous viviparous female **A** dorsal view of body **B** dorsal view of head **C** antenna **D** ultimate rostral segment **E** hind tibia and tarsi **F** marginal processes of thoracic nota and abdominal tergites I–IV **G** oval sculptures of abdominal tergites **H** siphunculus **I** irregular wavy sculptures of abdominal tergite VIII **J** cauda **K** anal plate **L** genital plate. Scale bars: 0.10 mm.

Figs 6 , 7 , Table 2

#### Specimens examined.

***Holotype***: apterous viviparous female, China: Shaanxi (Ankang City, 33.64°N, 109.37°E, altitude 2020 m), 16.VII.2017, No. 41008-1-1-1, on *Salix* sp., coll. H. Long and J.F. Ji. ***Paratypes***: one apterous viviparous female (slide), No. 41008-1-1-2 and one apterous viviparous female (COI: OK668436), two apterous viviparous females, No. 41029-1-1, with the same collection data as holotype; one apterous viviparous female, Shaanxi (Ankang City), 15.VII.2017, host plant unknown, No. 41000-1-1, coll. H. Long and J.F. Ji; two apterous viviparous females (slides), Shaanxi (Ankang City), 16.VII.2017, No. 41027-1-1 and one apterous viviparous female (COI: OK668437), host plant unknown, coll. H. Long and J.F. Ji (NHMUK).

#### Diagnosis.

Dorsum of body with oval sculptures; median frontal tubercle protuberant, hemispherical, antennal tubercles each with a short finger-shaped process at inner apex, lower than median frontal tubercle; meso-, metanotum, and abdominal tergites I–IV each with one pair of strongly imbricated and long horn-shaped marginal processes; dorsal setae of abdomen long and thick, slightly swollen at apices, with distinct setal tubercles.

#### Description.

Apterous viviparous females: body elongated oval (Fig. 7A), yellowish white in life.

**Mounted specimens.** Body pale; distal part of rostrum, cauda and anal plate pale brown, other parts pale in color (Fig. 7A). See Table 2 for general measurements.

***Head*.** Ocular tubercles small. Dorsum of head covered with semicircular and wavy sculptures on median area, marginal area smooth, venter with slight wrinkles (Figs 6A, 7B). Median frontal tubercle distinctly protuberant, rectangular (Figs 6A, 7B), with one pair of long, capitate setae on venter. Antennal tubercles undeveloped, each with a short finger-shaped process at inner apex, lower than median frontal tubercle (Figs 6A, 7B), each process with a long, and capitate seta at apex. Dorsal setae of head long and thick, slightly swollen at apices, with distinct setal tubercles. Head with one pair of cephalic setae, one pair of dorsal setae between antennae, and two pairs of dorsal setae between compound eyes arranged transversely. Antennae 4-segmented, Ant. I and Ant. II smooth, Ant. III–VI with slight imbrication; Ant. I slightly projected at inner apex (Figs 6B, 7C). Antennal setae long, thin and capitate on Ant. I and Ant. II, short and blunt on Ant. III and Ant. IV; Ant. I–IV with 4, 1, 0, 1 (base)+1 (PT) setae, respectively; apex of PT with two or three setae. Primary rhinaria ciliated. Rostrum reaching between mid- and hind coxae; URS wedge-shaped (Figs 6D, 7D), with three pairs of primary setae, without accessory setae.

***Thorax*.** Pronotum with semicircular and wavy sculptures on spino-pleural area, marginal area with small papillate tubercles. Meso- and metanotum with oval sculptures on spinal area, pleura-marginal area with oval sculptures and small papillate tubercles. Meso- and metanotum each with one pair of strongly imbricated and long horn-shaped marginal processes (Figs 6C, 7F), each process with a short capitate seta at apex and a short capitate seta at base. Dorsal setae of thorax long and thick, slightly swollen at apices, with distinct setal tubercles; pronotum with one pair of spinal, pleural and marginal setae, respectively, meso- and metanotum each with one pair of spinal and pleural setae. Legs normal, smooth. Setae on legs short, pointed ventrally and short, capitate dorsally. Hind tibiae with a row of short, thick, and blunt setae dorsally on middle (Fig. 7E). First tarsal chaetotaxy: 3, 2, 2. Second tarsal segments slightly imbricated.

***Abdomen*.** Abdominal tergites I–VII with distinctly oval sculptures on spino-pleural area (Figs 6E, 7G), marginal area with small papillate tubercles; tergite VIII with irregular wavy sculptures (Fig. 7I). Abdominal tergites I–IV each with one pair of strongly imbricated and long horn-shaped marginal processes (Figs 6C, F, 7F), each process with a short capitate seta at apex. Dorsal setae of abdomen long and thick, slightly swollen at apices, with distinct setal tubercles (Fig. 6H); ventral setae short and pointed. Abdominal tergites I–IV each with one pair of spinal and pleural setae, tergite VII with 2–4 spino-pleural setae, tergite VIII with 7–9 setae. Spiracles reniform, open or closed, spiracular plates slightly swollen. SIPH long spoon-shaped, straight, broad at base, thin at middle, slightly swollen distally, with imbrications, obliquely truncated at tip, without flange (Figs 6G, 7H). Cauda long tongue-shaped, slightly constricted at middle, with spinulose imbrications and four setae (Figs 6I, 7J). Anal plate semicircular, spinulose (Figs 6J, 7K); with 7–12 setae. Genital plate transversely oval, with sparse spinules in transverse rows (Figs 6K, 7L); with two anterior setae and four setae along the posterior margin.

#### Etymology.

The species is named for its distinctly long horn-shaped marginal processes on meso-, metanotum, and abdominal tergites I–IV; the Latin neuter adjective *cornutum* means “horned”.

#### Taxonomic notes.

The new species resembles *A.longituberculatum* (Zhang, Zhong & Zhang) in meso-, metanotum and abdominal tergites I–IV each with one pair of long horn-shaped marginal processes; but differs from it as follows: antennal tubercles each with a short finger-shaped process at inner apex, lower than median tubercle (the latter: antennal tubercles each with a long horn-shaped process at inner apex and higher than median tubercle); antenna 4-segmented, 0.35–0.40 × as long as body length (the latter: antenna 5-segmented, 0.43–0.47 × as long as body length); pronotum without marginal processes, meso- and metanotum and abdominal tergites I–IV with distinctly long horn-shaped marginal processes, 0.211–0.325 mm, about as long as SIPH (the latter: pronotum with short conical marginal processes, meso- and metanotum and abdominal tergites I–IV with long horn-shaped marginal processes, 0.084–0.206 mm, shorter than SIPH); dorsal setae of body long and thick, slightly swollen at apices, with distinct bases (the latter: dorsal setae thin, short and capitate); abdominal tergite VIII with 7–9 setae (the latter: abdominal tergite VIII with two setae).

#### Host plant.

*Salix* sp.

#### Distribution.

China (Shaanxi).

#### Biology.

The species disperses on the undersides of leaves.

### ﻿ Aspidophorodon musaicum

Taxon classificationAnimaliaHemipteraAphididae

Qiao, 2015

1028B372-0B01-5A50-819F-32964052E6B6

Aspidophorodon (Aspidophorodon) musaicum Qiao: Chen et al. 2015: 557, 560.

#### Specimens examined.

Two apterous viviparous females (holotype and paratype), China: Sichuan (Meigu County, altitude 2600 m), 04.V.2005, No. 17257-1-4, host plant unknown, coll. X.L. Huang.

#### Comment.

*Aspidophoron* being neuter, the adjectival specific epithet is also neuter, so *musaicus* is revised as *musaicum*.

#### Host plant.

Unknown.

#### Distribution.

China: Tibet.

#### Biology.

The species colonizes the undersides of leaves of its host plant.

### ﻿ Aspidophorodon obtusum

Taxon classificationAnimaliaHemipteraAphididae

Qiao, 2015

2AD4351E-F1A2-526C-BEB7-51F18A75411A

Aspidophorodon (Aspidophorodon) obtusum Qiao: Chen et al., 2015: 557,563.

#### Specimens examined.

Two apterous viviparous females and four fundatrices (holotype and paratypes), China: Sichuan (Luding County, Minya Konka), 16.V.2009, No. 22562-1-3-1, on *Salix* sp., coll. X.M. Su; one apterous viviparous female (slide) and one apterous viviparous (COI: OK668441), Sichuan (Luding County, Minya Konka), 30.IX.2019, No. 47777-1-2, on *Cotoneaster* sp., coll. J.F. Ji.

#### Comments.

The species have been collected on *Salixcupularis* in May (Chen et al. 2015) and on *Cotoneaster* sp. in September. The population on *Cotoneaster* sp. is without marginal processes, which differs from ones on *Salixcupularis* in morphological characteristics. However, the DNA sequences of the samples from both host plants are aligned 100% and other characters are similar between the two populations; therefore, there are two different host plant morphs in this species.

*Aspidophoron* being neuter, the adjectival specific epithet is also neuter, so *obtusus* is revised as *obtusum*.

#### Host plant.

*Salixcupularis*, *Cotoneaster* sp. (Rosaceae).

#### Distribution.

China: Sichuan.

#### Biology.

The species colonizes the undersides of leaves of its host plants (Fig. 21G). The life cycle is unknown.

### ﻿ Aspidophorodon reticulatum

Taxon classificationAnimaliaHemipteraAphididae

Qiao & Xu sp. nov.

47A42684-123D-52A6-B163-7B125B5A9E18

http://zoobank.org/9A45D20A-071D-4EAA-8AA9-8ED1FA4867A7

**Figure 8.**
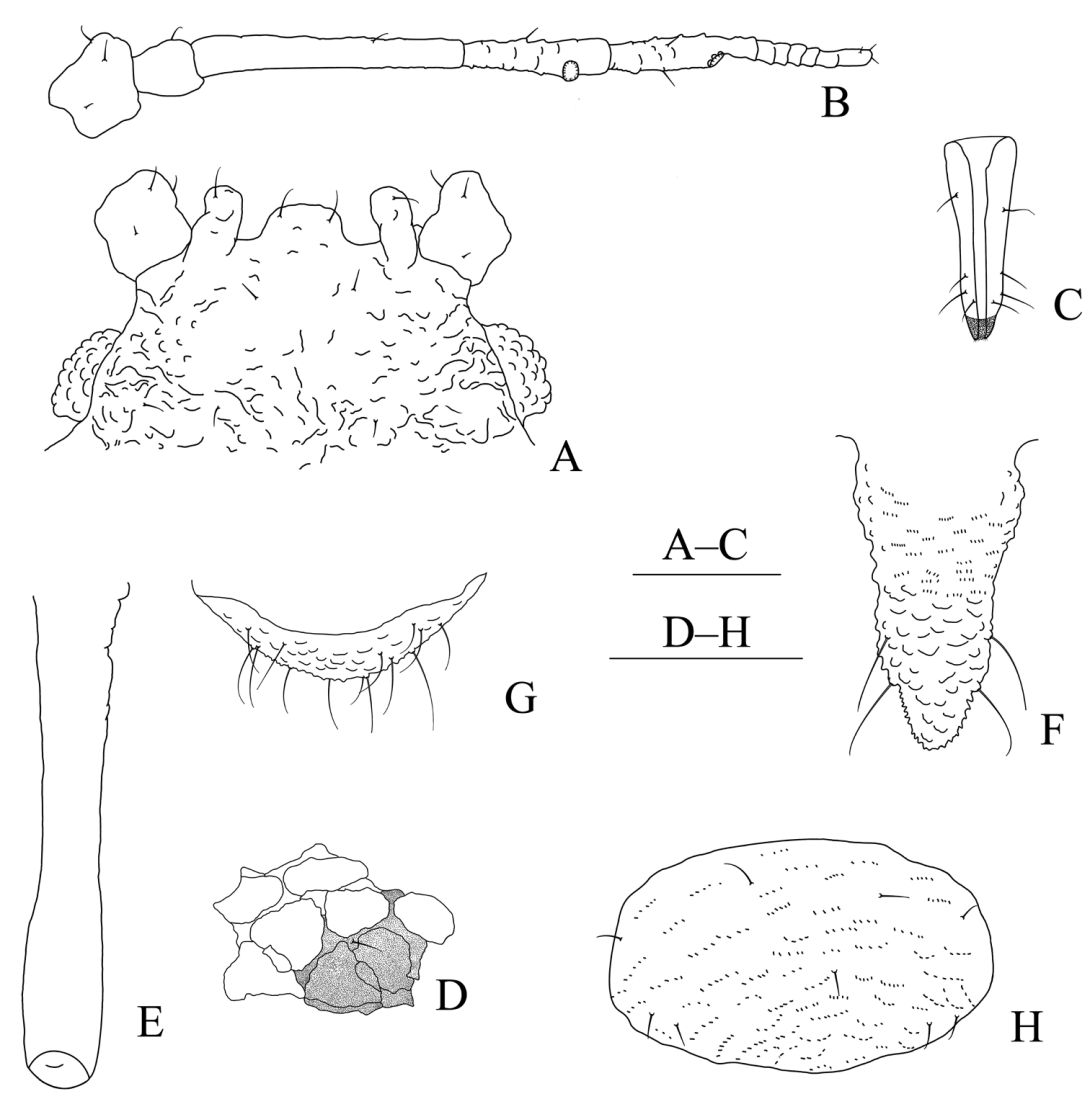
*Aspidophorodonreticulatum* Qiao & Xu, sp. nov. Apterous viviparous female **A** dorsal view of head **B** antenna **C** ultimate rostral segment **D** reticulations formed by strings of small triangles arranged in polygons on abdominal tergites **E** siphunculus **F** cauda **G** anal plate **H** genital plate. Scale bars: 0.10 mm.

**Figure 9.**
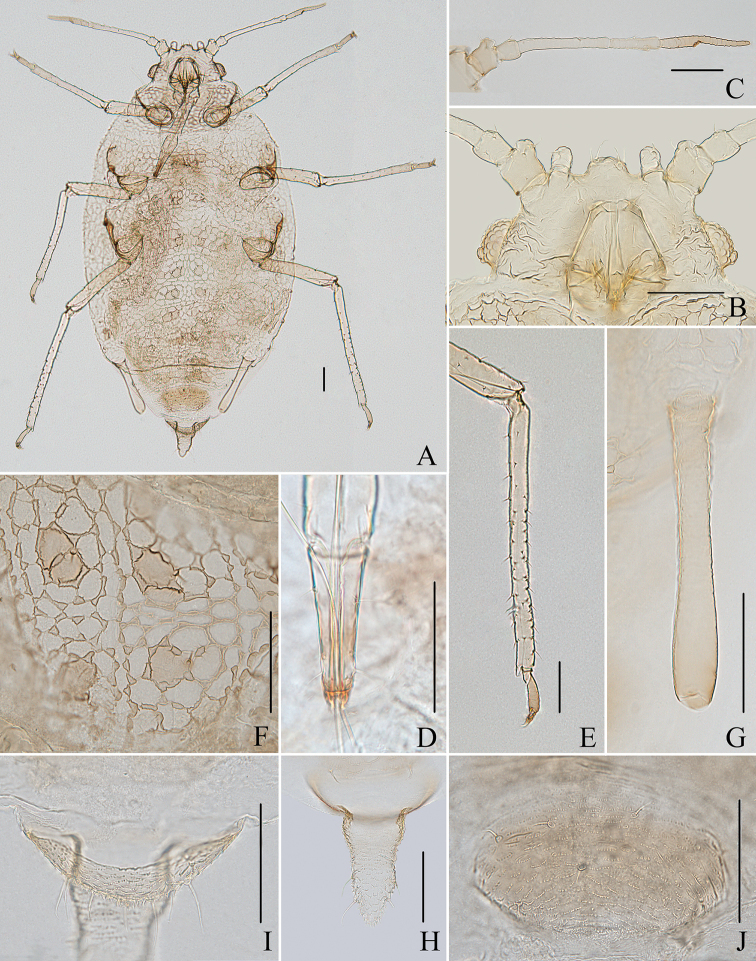
*Aspidophorodonreticulatum* Qiao & Xu, sp. nov. Apterous viviparous female **A** dorsal view of body **B** dorsal view of head **C** antenna **D** ultimate rostral segment **E** hind tibia and tarsi **F** reticulations formed by strings of small triangles arranged in polygons on abdominal tergites **G** siphunculus **H** cauda **I** anal plate **J** genital plate. Scale bars: 0.10 mm.

Figs 8 , 9 , Table 2

#### Specimens examined.

***Holotype***: apterous viviparous female, China: Tibet (Cuona County), 5.VI.2016, No. 37265-1-1, on *Salixcupularis*, coll. F. F. Niu; ***Paratypes***: one apterous viviparous female (slide) and one apterous viviparous female (COI: OK668435), No. 37265-1-2, with the same collection data as holotype.

#### Diagnosis.

Dorsum of body with distinct reticulations that consist of small triangles arranged in polygons; median frontal tubercle distinctly protuberant, rectangular; antennal tubercles each with a cylindrical process at inner apex, higher than median frontal tubercle; dorsal setae of abdomen sparse and short, with small setal tubercles.

#### Description.

Apterous viviparous females: body elliptical (Fig. 9A), green in life.

**Mounted specimens.** Body pale, PT, distal part of rostrum, tarsi, distal parts of SIPH, cauda, anal plate and genital plate pale brown (Fig. 9A). Thoracic nota and abdominal tergites I–VIII each with one pair of spinal sclerites, pale brown in color, other parts pale in color (Fig. 9A, F). See Table 2 for general measurements.

***Head*.** Ocular tubercles small. Dorsum of head covered with wrinkles between compound eyes, anterior part with weak wrinkles (Figs 8A, 9B). Median frontal tubercle distinctly protuberant, rectangular (Figs 8A, 9B), with one pair of long and pointed setae on venter. Antennal tubercles undeveloped, each with a cylindrical, slightly wrinkled process at inner apex, higher than median frontal tubercle, each process with a short and pointed seta at apex (Figs 8A, 9B). Dorsal setae of head short and slightly blunt, with small setal tubercles. Head with one pair of cephalic setae, one pair of dorsal setae between antennae and two pairs of dorsal setae between compounds eyes arranged transversely. Antennae 5-segmented, Ant. I–III smooth, Ant. IV–V with imbrications (Figs 8B, 9C); Ant. I slightly projected at inner apex. Antennal setae short and pointed, Ant. I–V with 3–4, 3, 1–2, 1, 1–2 (base) +1 (PT) setae, respectively; apex of PT usually with two setae. Primary rhinaria ciliated. Rostrum reaching mid-coxae; URS wedge-shaped (Figs 8C, 9D), with two pairs of primary setae and two pairs of accessory setae.

***Thorax*.** Thoracic nota with reticulations consist of small triangles arranged in polygons, those developed on pronotum. Dorsal setae of thorax short and blunt, with small setal tubercles; pronotum with two pairs of spinal setae, arranged anteriorly and posteriorly, one pair of pleural and one pair of marginal setae; meso- and metanotum each with one pair of spinal and one pair of pleural setae, two pairs of marginal setae. Legs normal, coxae and femora smooth, distal parts of tibiae imbricated. Setae on 2/3 distal part of femora and tibiae, short and pointed (Fig. 9E). First tarsal chaetotaxy: 3, 3, 2. Second tarsal segments with imbrications.

***Abdomen*.** Abdominal tergites I–VII with reticulations consisting of small triangles arranged in polygons (Figs 8D, 9F). Dorsal setae of abdomen sparse, short, pointed or slightly blunt, with small setal tubercles; ventral setae short and pointed. Abdominal tergites I–III each with one pair of spinal, pleural and marginal setae; tergites IV–VII each with one pair of spinal and one pair of marginal setae; tergites VIII with one pair of spinal setae. Spiracles reniform and open, spiracular plates slightly swollen. SIPH spoon-shaped, smooth, broad at base, thin at middle, swollen distally, obliquely truncated at tip, without flange (Figs 8E, 9G). Cauda elongate conical, slightly constricted at middle, with spinulose imbrications and four setae (Figs 8F, 9H). Anal plate semicircular, spinulose (Figs 8G, 9I), with 15–16 setae. Genital plate transversely oval, with sparse spinules in transverse rows (Figs 8H, 9J), with three or four anterior setae and four setae along the posterior margin.

#### Etymology.

The species is named for the reticulations apparent on the dorsum of the body, *reticulatum* being the neuter form of the adjective.

#### Taxonomic notes.

The new species resembles *A.harvense* Verma but differs from it as follows: antennal tubercles each with a cylindrical process at inner apex, 0.051–0.056mm, 1.82–2.04 × as long as its width (the latter: antennal tubercles each with a long finger-shaped process at inner apex, 0.110–0.120mm, 2.40–2.50 × as long as the basal width); dorsum of body with reticulations consist of small triangles arranged in polygons (the latter: dorsum of body with oval and irregular polygonal reticulations); URS 2.70–2.72 × as long as the basal width, 1.51–1.61 × as long as 2HT (the latter: URS 1.39–1.63 × as long as the basal width, 0.69–0.81 × as long as 2HT).

#### Host plant.

*Salixcupularis*.

#### Distribution.

China: Tibet.

#### Biology.

The species colonizes the undersides of leaves of its host plant.

### ﻿ Aspidophorodon salicis

Taxon classificationAnimaliaHemipteraAphididae

Miyazaki, 1971

E66776FA-3066-59D2-9383-AFC4A17EEF82

Aspidophorodon salicis Miyazaki 1971: 183; Eastop and Hille Ris Lambers 1976: 96; Blackman and Eastop 1994: 569.
Aspidophorodon sinisalicis Zhang: Zhang and Zhong 1980: 58.
Trichosiphonaphis lijiangensis Zhang, Zhong and Zhang 1992: 389.
Aspidophorodon (Aspidophorodon) salicis Miyazaki: Remaudière and Remaudière 1997: 74; Stekolshchikov and Novgorodova 2010: 44; Chen et al. 2015: 567.

#### Specimens examined.

Two alate viviparous females and 13 apterous viviparous females, China: Yunnan (Lijiang City), 27.V.1980, No. 7165, on *Salix* sp., coll. T.S. Zhong and L.Y. Wang; two apterous viviparous females, China: Gansu (Minxian County), 16.X.1985, No. 8326-1-4, on Salixmatsudanavar.tortuosa, coll. J.H. Li; four apterous viviparous females, China: Xinjiang (Burqin County), 23.VII.2007, No. 20604, host plant unknown, coll. D. Zhang; four apterous viviparous females, China: Ningxia (Jingyuan County, Mt. Liupan, altitude 1984 m), 26.VI.2008, No. 21540, on *Salix* sp., coll. J. Chen; one apterous viviparous female and one alate viviparous female, China: Sichuan (Leshan City), 12.VI.2009, No. 23167, on *Salix* sp., coll. J. J. Yu and X. Y. Li; one apterous viviparous female, China: Beijing (Mt. Baihua), 24.VIII.2015, No. 35920-1-1, on *Salix* sp., coll. H. Long; one apterous viviparous female, China: Hebei (Mt. Wuling), 18.VII.2016, No. 37942-1-1, on *Salix* sp., coll. R.J. Zhang and S.F. Xu; two apterous viviparous females, China: Shaanxi (Ankang City), 16.VII.2017, No. 41014-1-1, on Salicaceae, coll. H. Long and J.F. Ji; one apterous viviparous female, China: Sichuan (Ganzi County), 18.VII.2017, No. 45762-1-1, on *Salix* sp., coll. J.F. Ji; one apterous viviparous female, China: Sichuan (Mianyang City), 21.VII.2017, No. 41297-1-1, on *Salix* sp., coll. C. Gao; two apterous viviparous females (slides) and one apterous viviparous female (COI: OK668443), China: Hebei (Mt. Xiaowutai), 6.V.2021, No. 49999-1-1, on *Salix* sp., coll. Y. Xu.

#### Host plant.

*Polygonum* sp., *Salixpseudotangii*, *Salixudensis*, *Salix* sp.

#### Distribution.

China (Beijing, Gansu, Ningxia, Sichuan, Shaanxi, Xinjiang, Yunnan), Japan, Russia (Sakhalin and the Kuril Islands).

#### Biology.

This species colonizes the undersides of leaves of its host plant (Fig. 21H–J).

### ﻿Subgenus Eoessigia

Taxon classificationAnimaliaHemipteraAphididae

David, Rajasingh & Narayanan, 1972

23ECB20D-1F8A-5D96-B574-08237BFAFA1A

Eoessigia David, Rajasingh & Narayanan 1972: 35. Type species: Eoessigiaindicum David, Rajasingh & Narayanan 1972; by original designation.
Indotuberoaphis Chakrabarti & Maity 1984: 198. Type species: Indotuberoaphissorbi Chakrabarti & Maity 1984; by original designation.
Margituberculatus Zhang, Zhong & Zhang 1992: 381. Type species: Margituberculatuslongituberculatum Zhang, Zhong & Zhang 1992; by original designation.
Raychaudhuriella Chakrabarti 1978: 355. Type species: RaychaudhuriellamyzaphoidesChakrabarti 1978: 357; by original designation.
Aspidophorodon (Eoessigia) David, Rajasingh & Narayanan: Remaudière and Remaudière 1997: 74; Stekolshchikov and Novgorodova 2010: 44; Nieto Nafría et al. 2011: 198; Chen et al. 2015.

#### Comments.

Spinal processes at least present on abdominal tergite VIII; antennal tubercles each with an inconspicuous or finger-shaped process at inner apex; antenna 4–6 segmented; cauda with 4–11 setae; mainly on Rosaceae, sometimes on Salicaceae.

The subgenus contains eight species, including three new species. Aspidophorodon (Eoessigia) indicum (David, Rajasingh & Narayanan) is first recorded in China. *Aspidophorodoncornuatum* Qiao, 2015 is considered as a junior synonym of *Aspidophorodonlongituberculatum* (Zhang, Zhong & Zhang, 1992) syn. nov., as discussed below. Species of this subgenus occur in Canada, China, India, and Russia (the Altai Republic).

### ﻿Key to species of subgenus Eoessigia (based on apterous female viviparous)

**Table:** 

1	SIPH shorter than cauda; PT more than 1.5 × as long as base of the segment	***A.longicauda* (Richards)**
–	SIPH longer than cauda; PT shorter than 1.5 × as long as base of the segment	**2**
2	Thoracic nota and abdominal tergites I–IV each with 1 pair of marginal processes	**3**
–	Thoracic nota and abdominal tergites I–IV without marginal processes	**4**
3	Antennal tubercles each with a long finger-shaped process at inner apex; dorsum of head, thoracic nota and all abdominal tergites with paired or unpaired spinal processes; thoracic nota and abdominal tergites I–IV each with 1 pair of marginal processes	***A.sorbi* (Chakrabarti & Maity)**
–	Antennal tubercles each with a long horn-shaped process at inner apex; dorsum of head, thoracic nota and all abdominal tergites without spinal processes; thoracic nota and abdominal tergites I–IV each with 1 pair of long horn-shaped marginal processes	***A.longituberculatum* (Zhang, Zhong & Zhang)**
4	Spinal processes present on abdominal tergites VII–VIII	***A.vera* Stekolshchikov & Novgorodova**
–	Spinal processes only present on abdominal tergite VIII	**5**
5	Median frontal tubercle strongly depressed at middle into two cylinders; abdominal tergite VIII produced caudad into triangular process	**6**
–	Median frontal tubercle slightly depressed at middle; abdominal tergite VIII with conical spinal processes	**7**
6	Antennal tubercles each with a long finger-shaped process at inner apex, 0.077–0.095mm, higher than median frontal tubercle; rostrum reaching mid-coxae, URS 2.21–3.18 × as long as its width, 1.31–1.62 × as long as 2HT; triangular spinal processes on abdominal tergite VIII blunt at apex	***A.furcatum* Qiao & Xu, sp. nov.**
–	Antennal tubercles each with a short finger-shaped process at inner apex, 0.027–0.047mm, as high as median frontal tubercle; rostrum reaching meta-coxae, URS 3.28–3.42 × as long as its width, 1.56–1.92 × as long as 2HT; triangular spinal processes on abdominal tergite VIII constricted at apex	***A.longirostre* Qiao & Xu, sp. nov.**
7	Antenna 5-segmented, 0.30–0.36 × as long as body length; antennal tubercles each with a weakly protuberant process at inner apex and slightly lower than median frontal tubercle; dorsum of head with distinct wrinkles between compound eyes, thoracic nota and abdominal tergites I–VII with slight wrinkles	***A.obtusirostre* Qiao & Xu, sp. nov.**
–	Antenna 6-segmented, 0.38–0.52 × as long as body length; antennal tubercles each with a short finger-shaped process at inner apex and lower than median frontal tubercle; dorsum of head with densely semicircular and wavy sculptures, thoracic nota and abdominal tergites I–VII with semicircular and wavy sculptures	***A.indicum* (David, Rajasingh & Narayanan)**

### ﻿ Aspidophorodon (Eoessigia) furcatum

Taxon classificationAnimaliaHemipteraAphididae

Qiao & Xu sp. nov.

9621701A-64DA-5227-8569-04D9BB6C8382

http://zoobank.org/7BFCA822-1A5A-469E-8BD6-CE4531EA88AF

**Figure 10.**
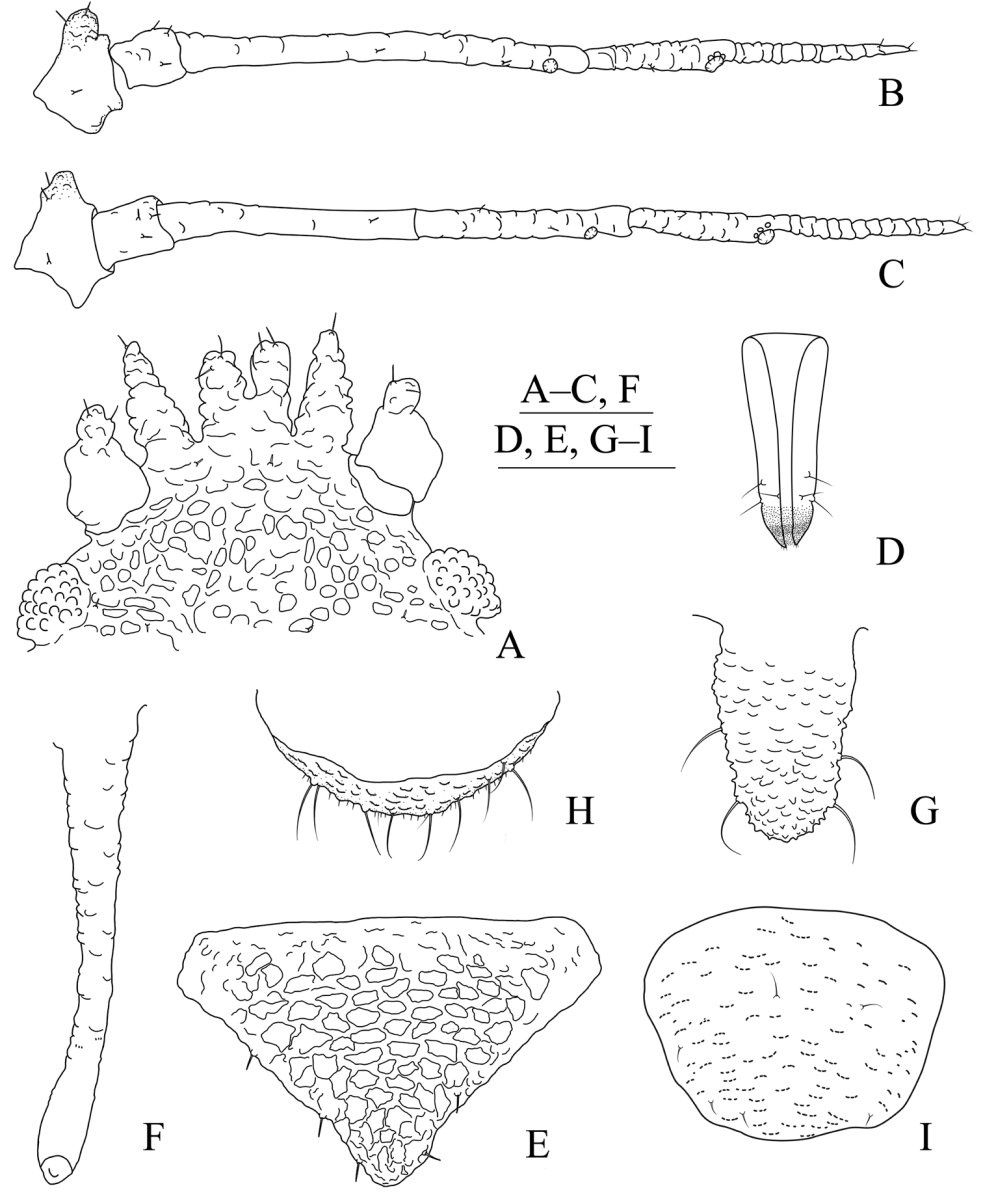
Aspidophorodon (Eoessigia) furcatum Qiao & Xu, sp. nov. Apterous viviparous female **A** dorsal view of head **B** antenna 4-segmented **C** antenna 5-segmented **D** ultimate rostral segment **E** spinal process of abdominal tergite VIII **F** siphunculus **G** cauda **H** anal plate **I** genital plate. Scale bars: 0.10 mm.

**Figure 11.**
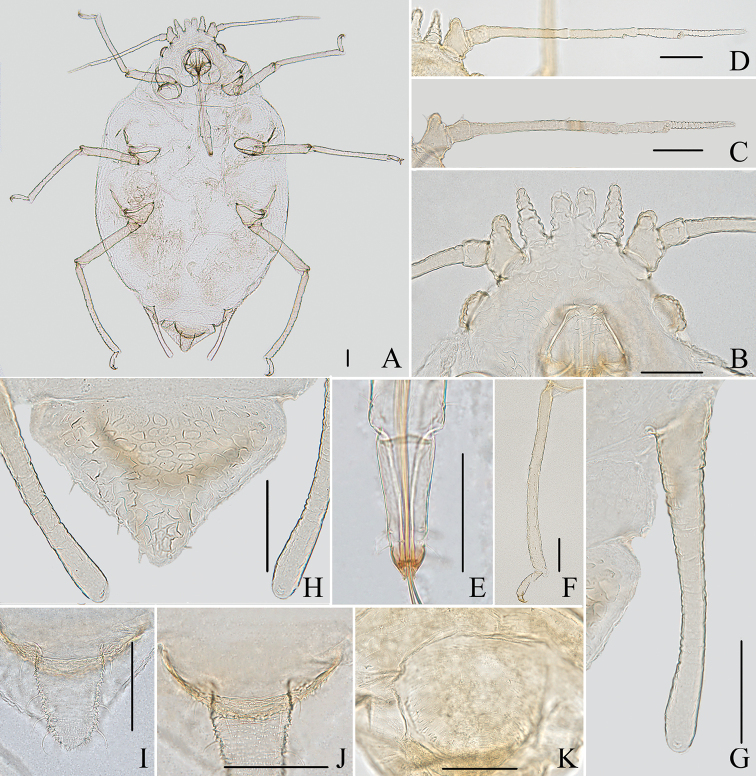
Aspidophorodon (Eoessigia) furcatum Qiao & Xu, sp. nov. Apterous viviparous female **A** dorsal view of body **B** dorsal view of head **C** antenna 4-segmented **D** antenna 5-segmented **E** ultimate rostral segment **F** hind tibia **G** siphunculus **H** spinal process of abdominal tergite VIII **I** cauda **J** anal plate **K** genital plate. Scale bars: 0.10 mm.

**Figure 12.**
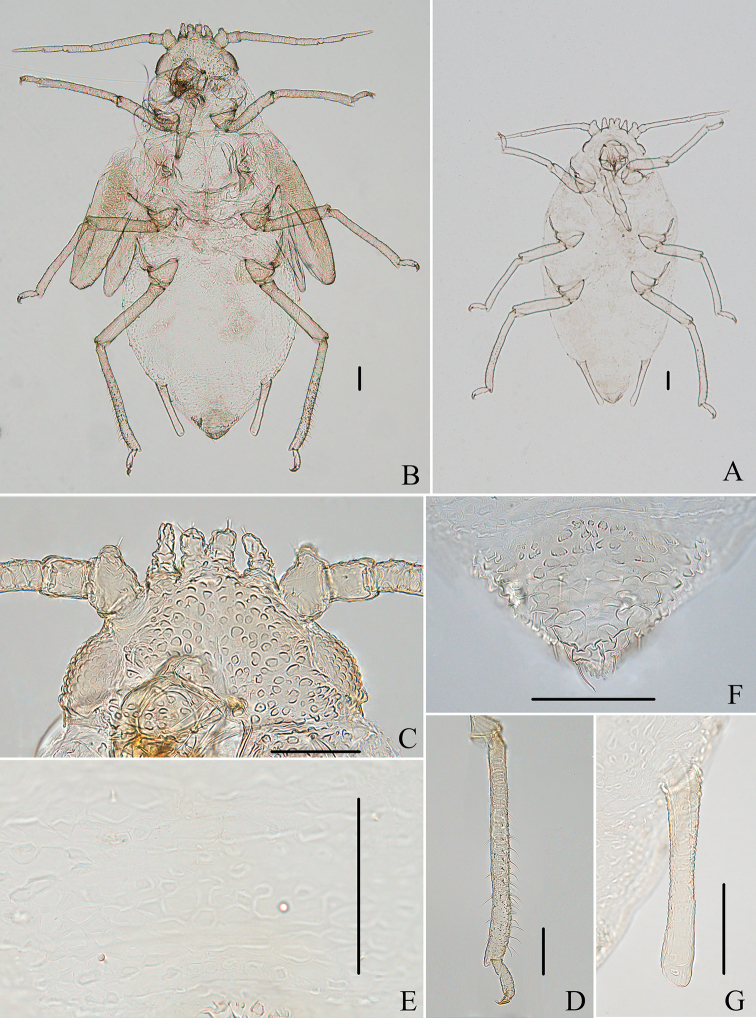
Aspidophorodon (Eoessigia) furcatum Qiao & Xu, sp. nov. **A** dorsal view of fourth instar apterous nymph. Fourth instar alate nymph **B** dorsal view of body **C** dorsal view of head **D** hind tibia and tarsi **E** oval and wavy sculptures of abdomen tergites **F** spinal process of abdominal tergite VIII **G** siphunculus. Scale bars: 0.10 mm.

Figs 10 , 11 , 12 , 22A, B , Table 3

#### Specimens examined.

***Holotype***: apterous viviparous female, China: Sichuan (Ganzi City, Minya Konka, 29.55°N, 101.97°E, altitude 3617 m), 25.VII.2019, No. 45915-1-1, on *Salix* sp., coll. J.F. Ji. ***Paratypes***: five apterous viviparous females (slides) and one apterous viviparous female (COI: OK668439), No. 45911-1-1, with the same collection data as holotype; two apterous viviparous females (slides) and one apterous viviparous female (COI: OK668438), China: Sichuan (Luding County, Minya Konka), 20.VII.2019, No. 45884-1-1, on *Salix* sp., coll. J.F. Ji; one apterous viviparous female, China: Sichuan (Luding County, Minya Konka), 22.VII.2019, No. 45896-1-1, on *Salix* sp., coll. J.F. Ji (NHMUK); one fourth instar apterous nymph, China: Sichuan (Luding County, Minya Konka), 23.VII.2019, No. 45906-1-1, on *Salix* sp., coll. J.F. Ji; three fourth instar alate nymphs, China: Sichuan (Luding County, Minya Konka), 27.IX.2019, No. 47741, on *Salix* sp., coll. J.F. Ji; three fourth instar alate nymphs, China: Sichuan (Luding County, Minya Konka), No. 47737-1-2, on *Salix* sp., coll. J.F. Ji, two fourth instar alate nymphs China: Tibet (Linzhi City, 29.57°N, 94.57°E, altitude 3550 m), 30.VIII.2020, No. 49104-1-1, on *Salix* sp., coll. Y. Xu.

#### Diagnosis.

Head dorsum covered with oval and wavy sculptures; median frontal tubercle well-developed, strongly imbricated, with a strong depression at middle separating it into two cylinders, hence fork-shaped; antennal tubercles each with a long finger-shaped and strongly imbricated process at inner apex, higher than median frontal tubercle; abdominal tergite VIII produced caudad into triangular spinal process which reaches the end of the cauda and covered with distinctly irregular polygonal markings and marginal area with wavy sculptures.

#### Description.

Apterous viviparous females: body broadly oval (Fig. 11A), yellowish in life (Fig. 22A, B).

**Mounted specimens.** Body pale in color (Fig. 11A). See Table 3 for general measurements.

**Table 3.** Morphometric data about species of the subgenus Aspidophorodon (Eoessigia) (in mm).

Parts		*A.furcatum* Qiao & Xu, sp. nov.			*A.indicum* (David, Rajasingh & Narayanan)			*A.longirostre* Qiao & Xu, sp. nov.		*A.obtusirostre* Qiao & Xu, sp. nov.
		Apterous viviparous female (n = 8)	4^th^ apterous nymph (n = 1)	4^th^ alate nymph (n = 2)	Apterous viviparous female (n = 10)	Alate viviparous female (n = 1)	Fundatrice (n = 2)	Apterous viviparous female (n = 3)	4^th^ alate nymph (n = 1)	Apterous viviparous female (n = 8)
Length (mm)	Body length	1.969–2.218	1.519	1.623–1.728	1.740–2.528	1.861	1.717–1.790	1.136–1.487	1.076	1.102–1.468
	Body width	0.997–1.166	0.716	0.720–0.746	0.721–1.041	0.644	0.730–0.770	0.514–0.708	0.508	0.600–0.714
	Antennae	0.646–0.766	0.566	0.688–0.692	0.752–1.193	/	0.645–0.660	0.451–0.568	0.296	0.389–0.484
	Ant. I	0.078–0.082	0.064	0.065–0.070	0.075–0.099	0.090	0.087–0.089	0.047–0.059	0.035	0.054–0.059
	Ant. II	0.042–0.055	0.045	0.057–0.059	0.046–0.060	0.063	0.046–0.047	0.033–0.037	0.034	0.034–0.040
	Ant. III	0.164–0.305	0.244	0.152–0.158	0.171–0.324	0.410	0.239	0.185–0.259	0.160	0.093–0.144
	Ant. IV	0.127–0.162	/	0.079–0.094	0.105–0.221	0.191	0.094–0.097	/	/	0.056–0.080
	Ant. IVb	/	0.092	/	/	/	/	0.055–0.087	0.067	/
	Ant. V	/	/	0.112–0.015	0.121–0.201	/	/	/	/	0.074–0.084
	Ant. Vb	0.093–0.120	/	0.085–0.088	/	/	0.096–0.097	/	/	/
	Ant. VIb	/	/	/	0.101–0.143	/	/	/	/	/
	PT	0.113–0.166	0.121	0.122–0.124	0.119–0.165	/	0.082–0.092	0.119–0.132	0.101	0.076–0.089
	URS	0.115–0.129	0.107	0.104–0.112	0.096–0.132	0.118	0.094–0.100	0.123–0.128	0.122	0.054–0.066
	Hind femur	0.346–0.412	0.275	0.296–0.310	0.341–0.556	0.505	0.328–0.339	0.207–0.297	0.193	0.197–0.299
Length (mm)	Hind tibia	0.577–0.680	0.463	0.464–0.481	0.641–1.015	0.992	0.547–0.567	0.366–0.480	0.337	0.333–0.425
	2HT	0.071–0.094	0.078	0.072–0.077	0.104–0.124	0.117	0.080–0.083	0.064–0.082	0.071	0.071–0.088
	SIPH	0.325–0.415	0.251	0.214–0.237	0.314–0.432	0.271	0.398–0.401	0.240–0.294	0.210	0.024–0.028
	BW SIPH	0.047–0.059	0.047	0.041–0.042	0.059–0.076	0.033	0.050–0.062	0.039–0.048	0.039	0.046–0.052
	MW SIPH	0.024–0.032	0.023	0.025–0.040	0.020–0.026	0.018	0.033–0.041	0.016–0.018	0.018	0.017–0.021
	DW SIPH	0.030–0.036	0.031	0.031–0.036	0.025–0.030	0.029	0.024–0.026	0.022–0.027	0.024	0.019–0.025
	Cauda	0.093–0.128	/	/	0.179–0.246	0.106	0.167–0.170	0.084–0.095	/	0.161–0.129
	BW Cauda	0.074–0.103	/	/	0.100–0.134	0.099	0.067–0.068	0.056–0.062	/	0.069–0.085
	MW Cauda	0.056–0.065	/	/	0.047–0.069	0.039	0.048–0.054	0.041	/	0.052–0.066
	Ant. IIIBD	0.027–0.038	0.039	0.031–0.034	0.026–0.033	0.033	0.028–0.030	0.017–0.022	0.021	0.019–0.022
	Widest width of hind femur	0.062–0.069	0.064	0.056–0.058	0.057–0.072	0.059	0.062–0.068	0.044–0.051	0.050	0.053–0.151
	MW hind tibia	0.033–0.040	0.044	0.037–0.041	0.029–0.037	0.027	0.034	0.024–0.027	0.031	0.023–0.029
	Cephalic setae	0.012–0.017	0.012	0.015–0.016	0.020–0.028	0.026	0.029–0.030	0.010–0.017	0.015	0.031–0.038
	Dorsal setae of head	0.007–0.010	0.007	0.006–0.007	0.004–0.006	0.011	/	0.005–0.007	0.006	/
	Dorsal setae of head between antennae	/	/	/	0.004–0.009	0.011	0.017–0.025	/	/	0.030–0.035
Length (mm)	Dorsal setae of head between compound eyes	/	/	/	0.004–0.008	0.009	0.010–0.012	/	/	0.009–0.011
	Marginal setae on Tergite I	0.006–0.010	0.004	0.005–0.006	0.004–0.007	0.010	0.008	0.004–0.008	0.003	0.008–0.009
	Spinal setae on Tergite VIII	0.015–0.021	0.016	0.014–0.026	0.018–0.025	0.017	0.024–0.026	0.013–0.018	0.016	0.032–0.040
	Setae on Ant. III	0.004–0.008	0.006	0.004–0.006	0.005–0.009	0.011	0.006–0.007	0.004–0.005	0.006	0.006–0.008
	Setae on hind tibia	0.577–0.680	0.463	0.464–0.681	0.017–0.026	0.021	0.015–0.017	0.020–0.022	0.038	0.018–0.026
	Processes on antennal tubercle	0.077–0.095	0.061	0.068–0.073	0.020–0.029	/	/	0.027–0.047	0.028	/
	Median frontal tubercle	0.063–0.077	0.054	0.055–0.064	/	/	/	0.025–0.046	0.024	/
	Marginal process on pronotum	/	/	/	/	/	0.064	/	/	/
	Marginal process on mesonotum	/	/	/	/	/	0.127–0.141	/	/	/
	Marginal process on metanotum	/	/	/	/	/	0.133–0.165	/	/	/
	Marginal process on Tergite I	/	/	/	/	/	0.192–0.206	/	/	/
	Marginal process on Tergite II	/	/	/	/	/	0.195–0.196	/	/	/
	Marginal process on Tergite III	/	/	/	/	/	0.175–0.208	/	/	/
	Marginal process on Tergite IV	/	/	/	/	/	0.201–0.224	/	/	/
	Spinal process on Tergite VIII	0.161–0.175	0.847	/	/	/	0.209	0.112–0.139	0.117	0.022–0.061
Ratio (times)	Body length / Body width	1.84–2.02	2.12	2.25–2.32	2.22–2.56	2.89	2.23–2.45	2.16–2.21	2.12	1.84–2.27
	Whole antennae / Body	0.31–0.36	0.37	0.40–0.42	0.38–0.52	/	0.36–0.38	0.37–0.40	0.37	0.30–0.35
	Hind femur / Ant. III	1.13–2.20	1.23	1.87–2.04	1.34–2.07	1.23	1.37–1.42	1.05–1.20	1.21	1.45–2.23
Ratio (times)	Hind tibia / Body	0.28–0.31	0.31	0.28–0.29	0.37–0.43	0.53	0.31–0.33	0.31–0.32	0.31	0.24–0.30
	Ant. I / Ant. III	0.23–0.50	0.26	0.43–0.44	0.28–0.44	0.22	0.36–0.37	0.22–0.25	0.22	0.38–0.59
	Ant. II / Ant. III	0.15–0.30	0.18	0.36–0.39	0.18–0.27	0.15	0.19–0.20	0.14–0.18	0.21	0.26–0.37
	Ant. IV / Ant. III	0.66–0.88	/	0.50–0.62	0.47–0.70	0.47	0.39–0.41	/	/	0.40–0.67
	Ant. V/ Ant. III	/	/	0.73–0.74	0.55–0.71	/	/	/	/	/
	Ant. IVb, Vb or VIb / Ant. III	0.35–0.58	0.38	0.54–0.58	0.37–0.62	/	0.40–0.41	0.30–0.35	0.42	0.51–0.81
	PT / Ant. III	0.45–0.78	0.50	0.79–0.80	0.40–0.78	/	0.34–0.39	0.48–0.71	0.63	0.62–0.82
	PT / Ant. IVb, Vb or VIb	1.10–1.57	1.32	1.39–1.46	0.93–1.34	/	0.85–0.95	1.37–2.38	1.51	0.98–1.25
	URS / BW URS	2.21–3.18	2.43	2.61–2.67	2.06–2.54	2.81	2.19–2.38	3.28–3.42	3.30	1.27–1.94
Ratio (times)	URS / 2HT	1.31–1.62	1.37	1.44–1.45	0.89–1.10	1.01	1.13–1.25	1.56–1.92	1.72	0.70–0.84
	SIPH / Body	0.16–0.19	0.17	0.13–0.14	0.16–0.21	0.15	0.22–0.23	0.20–0.21	0.20	0.16–0.23
	SIPH / Cauda	2.62–3.88	/	/	1.70–2.05	2.56	0.34–0.40	2.86–3.09	/	1.51–1.93
	SIPH / BW SIPH	5.70–7.62	5.34	5.10–5.78	5.23–6.44	8.21	6.42–8.02	6.13–6.69	5.39	4.88–5.51
	SIPH / MW SIPH	10.16–15.71	10.91	5.93–8.56	13.08–20.85	15.06	9.71–12.15	14.12–17.56	11.67	11.85–14.35
	SIPH / DW SIPH	9.03–12.57	8.10	5.94–7.65	11.63–14.60	9.35	15.31–16.71	10.89–12.22	8.75	10.36–12.84
	Cauda / BW Cauda	1.07–1.49	/	/	1.65–2.18	1.07	2.46–2.54	1.50–1.53	/	1.67–2.04
Ratio (times)	Cephalic setae / Ant. IIIBD	0.37–0.57	0.31	0.47–0.48	0.61–0.91	0.79	0.97–1.07	0.59–0.77	0.71	1.41–2.00
	Marginal setae on Tergite I / Ant. III BD	0.18–0.37	0.10	0.16–0.18	0.12–0.21	0.30	0.27	0.23–0.38	0.14	0.36–0.47
	Spinal setae on Tergite VIII / Ant. III BD	0.53–0.60	0.41	0.45–0.77	0.55–0.77	0.52	0.86–0.87	0.76–0.86	0.76	1.52–2.11
	Setae on Ant. III / Ant. IIIBD	0.13–0.28	0.15	0.13–0.17	0.17–0.28	0.33	0.20–0.25	0.18–0.29	0.29	0.27–0.38
	Setae on hind tibia / MW hind tibia	0.51–0.68	0.91	0.93–1.16	0.53–0.70	0.78	0.44–0.50	0.74–0.88	1.23	0.62–0.90
	Length of processes on antennal tubercle / its basal width	1.83–2.64	1.53	1.74–2.15	0.77–1.24	/	/	0.90–1.47	1.04	/
	Length of median frontal tubercle / its basal width	0.97–1.24	0.93	0.87–1.60	/	/	/	0.56–0.90	0.45	/
	Length of marginal process on pronotum / its basal width	/	/	/	/	1.94–2.00	/	/	/	/
	Length of marginal process on mesonotum / its basal width	/	/	/	/	2.59–3.07	/	/	/	/
	Length of marginal process on metanotum / its basal width	/	/	/	/	2.80–3.41	/	/	/	/
	Length of marginal process on Tergite I / its basal width	/		/	/	3.03–3.43	/	/	/	/
	Length of marginal process on Tergite II / its basal width	/			/	3.00–3.32	/	/	/	/
	Length of marginal process on Tergite III / its basal width	/	/	/	/	3.37–3.53	/	/	/	/
	Length of marginal process on Tergite IV / its basal width	/	/	//	/	3.24–3.25	/	/	/	/
	Length of spinal process on Tergite VIII / its basal width	0.49–0.67	0.85	/	/	/	/	0.62–0.76	1.05	0.19–0.38

***Head*.** Ocular tubercles small. Dorsum of head covered with oval and wavy sculptures, venter with wrinkles (Figs 10A, 11B). Median frontal tubercle well-developed, strongly imbricated, with a strong depression at the middle separating it into two cylinders, fork-shaped (Figs 10A, 11B), each cylinder with one pair of long and blunt setae at apex. Antennal tubercles undeveloped, each with a long finger-shaped, pointed, and strongly imbricated process at inner apex, higher than median frontal tubercle (Figs 10A, 11B), each process with a long and blunt seta at apex. Dorsal setae of head short and blunt, with small setal tubercles. Head with one pair of dorsal setae between antennae, and two pairs of dorsal setae between compound eyes arranged transversely. Antennae 4- or 5- segmented, Ant. I distinctly projected into short cylindrical at inner apex, 0.029–0.042mm, with two short and blunt setae at apex; Ant. I with slight wrinkles, other segments slightly imbricated (Figs 10B, C, 11C, D). Antennal setae short and blunt, Ant. I–V with 3 or 4, 3 or 4, 1 or 2, 1 or 2, 1–3 (base) +0 or 1 (PT) setae, respectively (or Ant. I–IV with 3 or 4, 3 or 4, 3 or 4, 2 or 3 (base) +1 (PT) setae, respectively), apex of PT with two or three setae. Primary rhinaria ciliated. Rostrum reaching mid-coxae, with apex pale brown; URS long wedge-shaped (Figs 10D, 11E), with three pairs of primary setae, and without accessory setae.

***Thorax*.** Pronotum with oval and wavy sculptures on spino-pleural area, marginal area with wrinkles. Meso- and metanotum with wrinkles on marginal area, spino-pleural area smooth. Thoracic setae sparse, short and blunt, with small setal tubercles; pronotum with two pairs of spinal setae, arranged anteriorly and posteriorly, one pair of pleural and one pair of marginal setae; meso- and metanotum each with one pair of spinal, one pair of pleural, and two pairs of marginal setae. Legs normal, short; coxae and femora smooth, distal parts of tibiae slightly imbricated. Setae on 2/3 distal part of femora and tibiae, short and blunt; hind tibiae with a row of short and blunt setae dorsally on the middle (Fig. 11F). First tarsal chaetotaxy: 3, 3, 2. Second tarsal segments slightly imbricated.

***Abdomen*.** Abdominal tergites I–VII with wrinkles on marginal area, spino-pleural area smooth; tergite VIII with irregular polygonal markings and marginal area with wavy sculptures, produced caudad into triangular spinal process reaching the end of the cauda (Figs 10E, 11H). Venter of abdominal tergites III–VIII with fine spinules arranged in rows. Dorsal setae of abdomen short and blunt, with small setal tubercles, ventral setae short and pointed. Abdominal tergites I and II each with one pair of spinal, one pair of pleural and one pair of marginal setae; tergites III–VII each with one pair of spinal and marginal setae; tergite VIII with five or six setae at margin. Spiracles reniform, open; spiracular plates slightly swollen. SIPH long spoon-shaped, incurved inward, broad at base, thin at the middle, slightly swollen distally, with imbrications, distal 1/4 smooth, obliquely truncated at tip, without flange (Figs 10F, 11G). Cauda wide tongue-shaped, slightly constricted at the middle, with spinulose imbrications and four or five setae (Figs 10G, 11I). Anal plate semicircular, spinulose (Figs 10H, 11J), with 11–14 setae. Genital plate broadly round, with sparse spinules in transverse rows (Figs 10I, 11K), with two anterior setae and four setae along the posterior margin.

**Fourth instar apterous nymph.** As in apterous viviparous females except as follows (Fig. 12A): legs normal; femora and tibia imbricated at distal part, hind tibia with numerous spinules and imbrications on 2/3 distal part. Setae on 2/3 distal part of femora and tibiae, short and blunt; hind tibiae with long pointed setae dorsally and short pointed setae ventrally, and with a row of short, thick, and blunt setae dorsally on the middle.

**Fourth instar alate nymph. Mounted specimens**: body elongated oval and body pale in color (Fig. 12B). See Table 3 for general measurements.

***Head*.** As in apterous viviparous females except as follows: dorsum of head with oval sculptures, more developed than apterous viviparous females (Fig. 12C). Antennae 6-segmented, Ant. I distinctly projected into short cylindrical at inner apex, 0.026–0.031mm. Antennae setae short and blunt, Ant. I–IV with 4, 3–4, 1–2, 2–1, 1, 2–3 (base) +0 (PT) setae, respectively. Primary rhinaria ciliated, Ant. III–V each with 20 or 21, 8, 8 immature round secondary rhinaria.

***Thorax*.** As in apterous viviparous females except as follows: pronotum with oval and wavy sculptures at anterior part, pleura-marginal area with wavy sculptures; meso- and metanotum with wrinkles at spinal area, pleura-marginal area with oval and wavy sculptures. Legs normal; femora imbricated at distal part, tibia scabrous and with imbrications, hind tibia with numerous spinules and imbrications on 2/3 distal part (Fig. 12D). Setae on legs short and pointed; hind tibiae with long pointed setae dorsally and short pointed setae ventrally, and with a row of short, thick and blunt setae dorsally on middle. First tarsal chaetotaxy: 3, 3, 3.

***Abdomen*.** As in apterous viviparous females except as follows: dorsal sculptures more developed than apterous viviparous females; abdominal tergites I–VII with oval and wavy sculptures (Fig. 12E), those developed on marginal area; tergites VIII produced caudad into triangular spinal process with irregular polygonal sculptures posteriorly and scaly sculptures anteriorly, marginal area with wavy sculptures (Fig. 12F).

#### Etymology.

The species is named for the median frontal tubercle with a strong depression at middle creating a fork, hence the neuter adjective *furcatum* in Latin.

#### Taxonomic notes.

The new species resembles *A.indicum* (David, Rajasingh & Narayanan) in head with three processes on front; dorsum of head covered with distinctly oval and wavy sculptures; abdominal tergite VIII with a spinal tubercle; but differs from it as follows: median frontal tubercle well-developed, strongly imbricated, with a strong depression at the middle separating it into two cylinders, fork-shaped; antennal tubercles each with a long finger-shaped, pointed and strongly imbricated process at inner apex, higher than median frontal tubercle (the latter: median frontal tubercle protuberant rectangular and slightly depressed at the middle; antennal tubercles each with a short cylindrical and blunt process at inner apex, lower than median frontal tubercle); abdominal tergite VIII produced caudad into triangular process (the latter: abdominal tergite VIII with conical spinal process); dorsum of head covered with distinctly oval and wavy sculptures (the latter: dorsum of head with densely semicircular and wavy sculptures).

The new species resembles *A.longirostre* Qiao & Xu, sp. nov. in having its median frontal tubercle well-developed, strongly imbricated, with a strong depression at the middle separating it into two cylinders; abdominal tergite VIII produced caudad into triangular spinal process; SIPH long spoon-shaped, incurved inward, obliquely truncated at tip, without flange; cauda wide tongue-shaped, slightly constricted at the middle; but differs from it as follows: median frontal tubercle well-developed, 0.063–0.077 mm; a long finger-shaped process at inner apex of antennal tubercles, 0.077–0.095 mm, higher than median frontal tubercle (the latter: median frontal tubercle protuberant, 0.025–0.046mm; a finger-shaped process at inner apex of antennal tubercles, 0.027-0.047, as high as median frontal tubercle); rostrum reaching mid-coxae, URS long wedge-shaped, 2.21–3.18 × as long as its width, 1.31–1.62 × as long as 2HT (the latter: rostrum reaching hind coxae, URS thin and long wedge-shaped, 3.28–3.42 × as long as its width, 1.56–1.92 × as long as 2HT); abdominal tergite VIII with irregular polygonal markings and marginal area with wavy sculptures, blunt at apex (the latter: abdominal tergite VIII with oval sculptures, constricted at apex).

#### Host plant.

*Salix* sp.

#### Distribution.

China (Sichuan, Tibet).

#### Biology.

This species colonizes the undersides of leaves of its host plant (Fig. 22A, B).

### ﻿ Aspidophorodon (Eoessigia) indicum

Taxon classificationAnimaliaHemipteraAphididae

(David, Rajasingh & Narayanan, 1972)

8FA16DB6-3664-551E-A950-23F3E169A0FA

**Figure 13.**
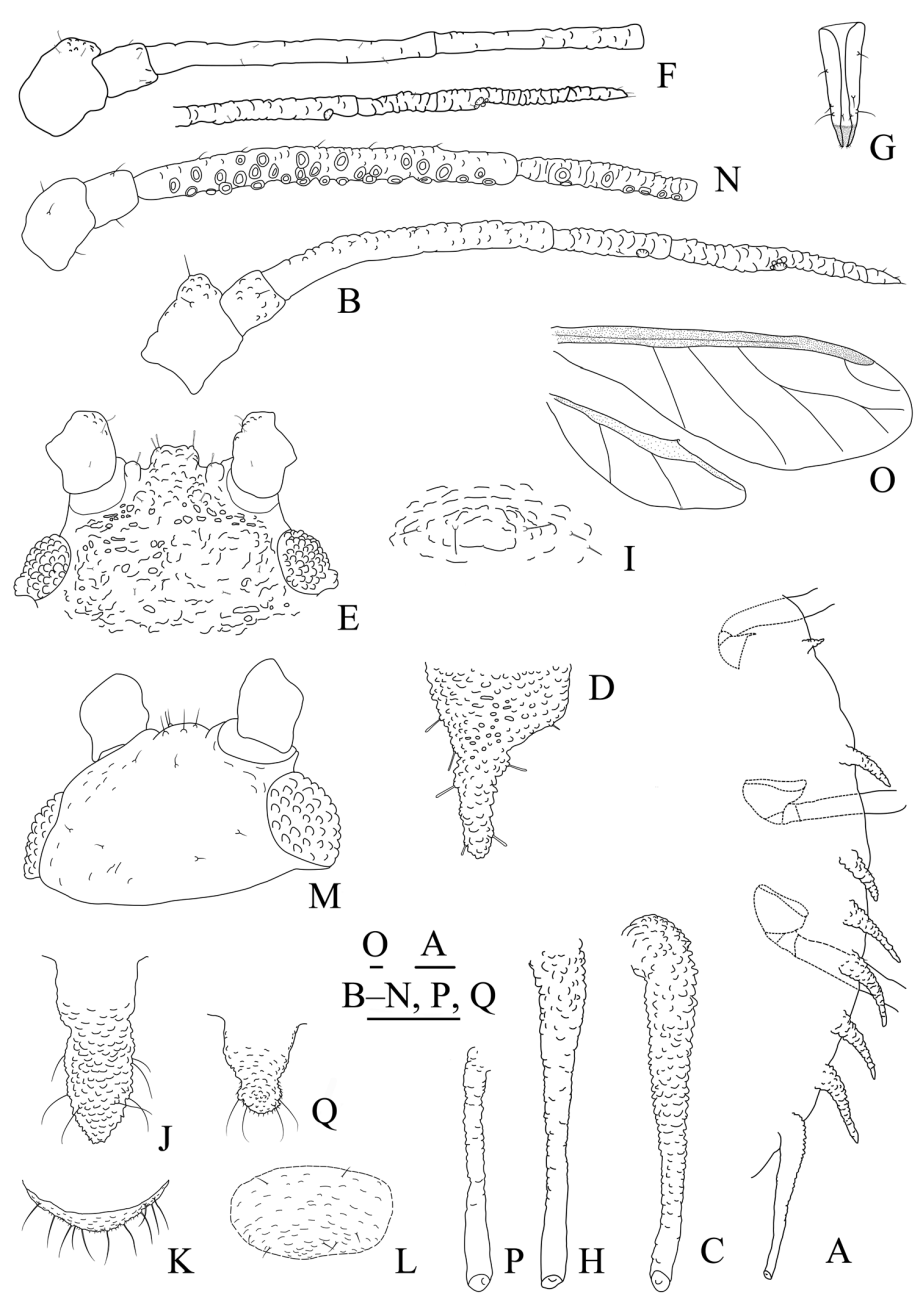
Aspidophorodon (Eoessigia) indicum (David, Rajasingh & Narayanan). Fundatrices **A** marginal processes of thoracic nota and abdominal tergites I–IV **B** antenna **C** siphunculus **D** spinal process of abdominal tergite VIII. Apterous viviparous female **E** dorsal view of head **F** antenna **G** ultimate rostral segment **H** siphunculus **I** spinal process of abdominal tergite VIII **J** cauda **K** anal plate **L** genital plate. Alate viviparous female **M** dorsal view of head **N** antennal segments I–IV **O** wings **P** siphunculus **Q** cauda. Scale bars: 0.10 mm.

**Figure 14.**
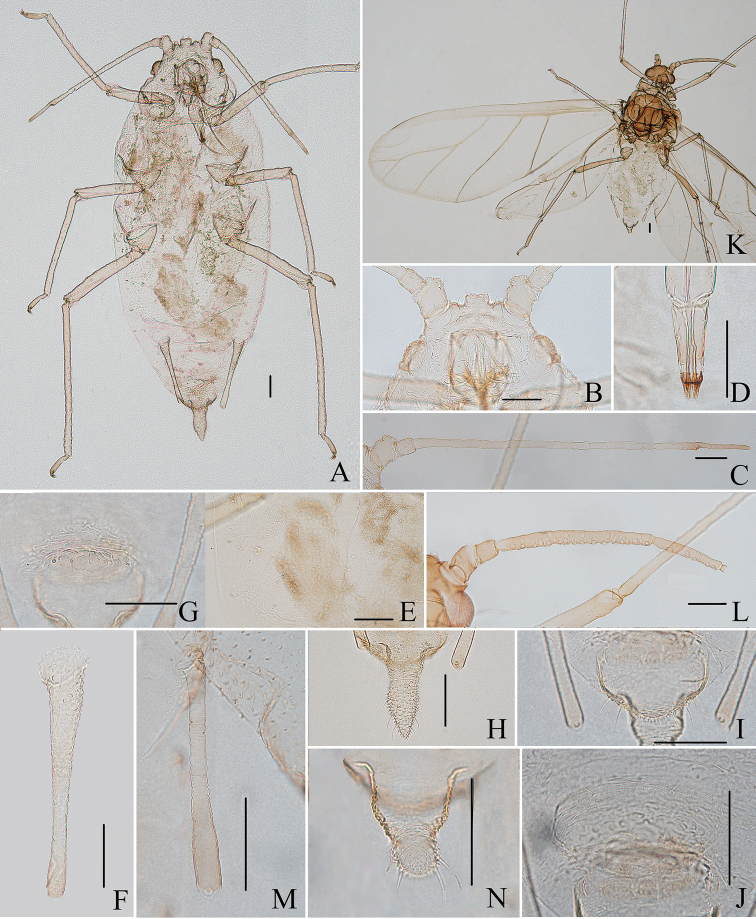
Aspidophorodon (Eoessigia) indicum (David, Rajasingh & Narayanan). Apterous viviparous female **A** dorsal view of body **B** dorsal view of head **C** antenna **D** ultimate rostral segment **E** sculptures of abdominal tergites **F** siphunculus **G** spinal process of abdominal tergite VIII **H** cauda **I** anal plate **J** genital plate. Alate viviparous female **K** dorsal view of body **L** antennal segments I–IV **M** siphunculus **N** cauda. Scale bars: 0.10 mm.

**Figure 15.**
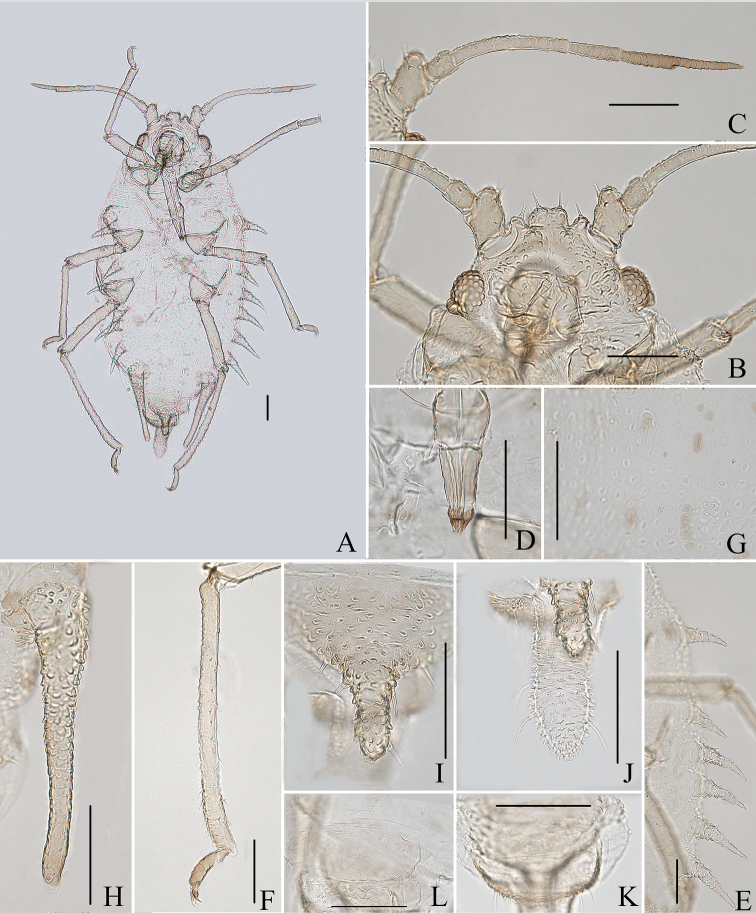
Aspidophorodon (Eoessigia) indicum (David, Rajasingh & Narayanan). Fundatrices **A** dorsal view of body **B** dorsal view of head **C** antenna **D** ultimate rostral segment **E** marginal processes of thoracic nota and abdominal tergites I–IV **F** hind tibia and tarsi **G** oval sculptures of abdominal tergites **H** siphunculus **I** spinal process of abdominal tergite VIII **J** cauda **K** anal plate **L** genital plate. Scale bars: 0.10 mm.

Figs 13 , 14 , 15 , 22C–F , Table 3

Eoessigia indicum David, Rajasingh & Narayanan 1972: 35; Eastop and Hille Ris Lambers 1976: 188; Chakrabarti and Medda 1989: 133.
Raychaudhuriella myzaphoides Chakrabarti 1978: 357.
Raychaudhuriella potentillae Chakrabarti & Maity 1984: 202.
Aspidophorodon (Eoessigia) indicum (David, Rajasingh & Narayanan): Remaudière and Remaudière 1997: 74; Blackman and Eastop 2006: 1098; Stekolshchikov and Novgorodova 2010: 44; Chen et al. 2015.

#### Specimens examined.

One apterous viviparous female, China: Tibet (Yadong County), 17.VII.2014, No. 32675-1-1, host plant unknown, coll. J. Chen and X.C. Zhu; four apterous viviparous females, China: Tibet (Cuona County), 01.VI.2016, No. 37202-1-1, No. 37204-1-1, No. 37205-1-1, No. 37208-1-1, on *Cotoneaster* sp., coll. F.F. Niu; two apterous viviparous females, China: Tibet (Cuona County), 03.VI.2016, No. 37225-1-1, No. 37232-1-1, on *Cotoneaster* sp., coll. F.F. Niu; one apterous viviparous female, China: Tibet (Cuona County), 04.VI.2016, No. 37243-1-1, on *Cotoneaster* sp., coll. F.F. Niu; two apterous viviparous females, China: Tibet (Cuona County), 07.VI.2016, No. 37278-1-1, No. 37280-1-1, on *Cotoneaster* sp., coll. F.F. Niu; one apterous viviparous female, China: Tibet (Cuona County), 24.VI.2016, No. 37403-1-1, on *Cotoneaster* sp., coll. F.F. Niu; two apterous viviparous females, China: Tibet (Cuona County), 03.VI.2016, No. 37229-1-1, No. 37230-1-1, host plant unknown, coll. F.F. Niu; one alate viviparous female, China: Tibet (Cuona County), 03.VI.2016, No. 37223-1-1, on *Cotoneaster* sp., coll. F.F. Niu; two apterous viviparous females (slides) and one apterous vivparous female (COI: OK668434), China: Tibet (Jilong County), 31.VII.2021, No. 52024-1-1, on *Cotoneaster* sp., coll. Y. Xu; two fundatrices (slides) and one fundatrix (COI: OK668447), China: Tibet (Jilong County), 01.VIII.2021, No. 52044-2-1, on *Cotoneaster* sp., coll. Y. Xu.

#### Comments.

The species is here first recorded in China. After several surveys in Tibet, we collected fundatrices (Figs 13A–D, 15A–L, 22F), apterous viviparous females (Figs 13E–L, 14A–J, 22C–E), and alate viviparous females (Figs 13M–Q, 14K–N) feeding on upper sides of *Cotoneaster* sp. along the main vein (Fig. 22C–F). The processes are variable in different morphs. Firstly, the marginal processes of thoracic nota and abdominal tergites I–IV (Figs 13A, 15E) and spinal process of abdominal tergite VIII (Figs 13D, 15I) are very developed in the fundatrix, but the apterae and alatae are without marginal processes, and spinal process of abdominal tergite VIII (Figs 13I, 14G) is shorter than that of the fundatrix. Secondly, about the different geographic populations of apterae, abdominal tergite VIII is with a distinctly triangular spinal process in a population from Jilong County; however, another population collected in Yadong County only has a slightly swollen spinal process.

*Aspidophoron* being neuter, the adjectival specific epithet is also neuter, so *indica* is revised as *indicum*.

#### Host plant.

Primary host plants: *Cotoneasterobtusus* and *Cotoneaster* sp.; secondary host plant: *Potentilla* sp. (Chakrabarti and Medda 1989).

#### Distribution.

China (Tibet), India.

#### Biology.

The species colonizes on upper sides of *Cotoneaster* sp. along the main vein (Fig. 22C–F). The species is holocyclic and heteroecious, alternating between *Cotoneaster* and *Potentilla* (Chakrabarti and Medda 1989), and colonizes the undersides of *Potentilla* without ant-attendance (Chakrabarti and Maity 1984).

### ﻿ Aspidophorodon (Eoessigia) longicauda

Taxon classificationAnimaliaHemipteraAphididae

(Richards, 1963)

C41C6630-6348-52BE-9C3A-A34410AEE126

Aspidaphis longicauda Richards 1963: 297.
Eoessigia longicauda Eastop & Hille Ris Lambers 1976: 95.
Aspidophorodon (Eoessigia) longicauda (Richards): Remaudière and Remaudière 1997: 74; Blackman and Eastop 2006: 1099; Stekolshchikov and Novgorodova 2010: 44.

#### Host plant.

*Spiraea* sp.

#### Distribution.

Canada.

#### Biology.

The species occurs on the under surfaces of leaves of *Spiraea* sp. (Richards, 1963).

### ﻿ Aspidophorodon (Eoessigia) longirostre

Taxon classificationAnimaliaHemipteraAphididae

Qiao & Xu sp. nov.

C8885893-F177-5A77-8AA1-D9DB5E3DC536

http://zoobank.org/FD1E0FEE-0054-4077-AA30-C90D6DA70956

**Figure 16.**
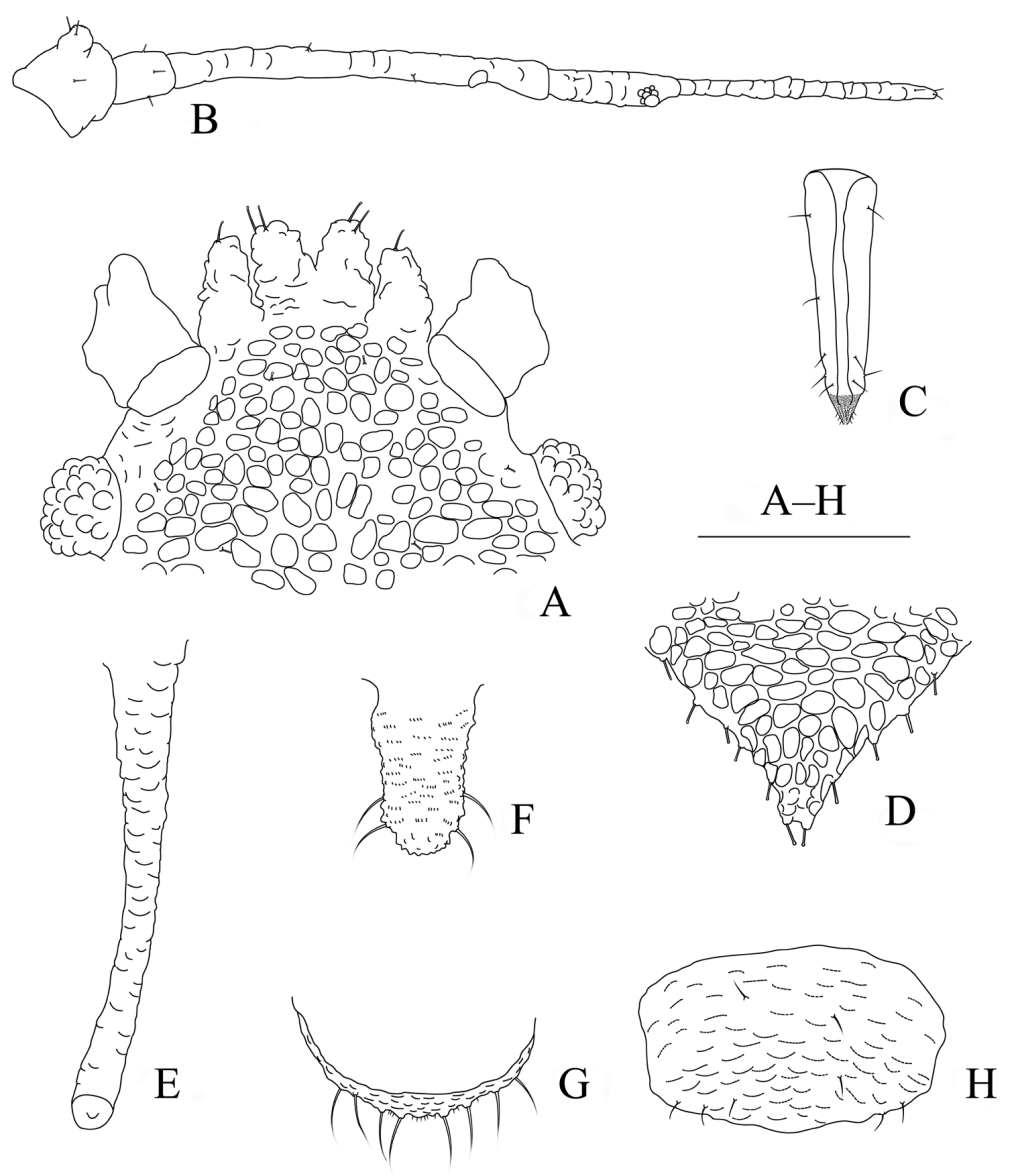
Aspidophorodon (Eoessigia) longirostre Qiao & Xu, sp. nov. Apterous viviparous female **A** dorsal view of head **B** antenna **C** ultimate rostral segment **D** spinal process of abdominal tergite VIII **E** siphunculus **F** cauda **G** anal plate **H** genital plate. Scale bars: 0.10 mm.

**Figure 17.**
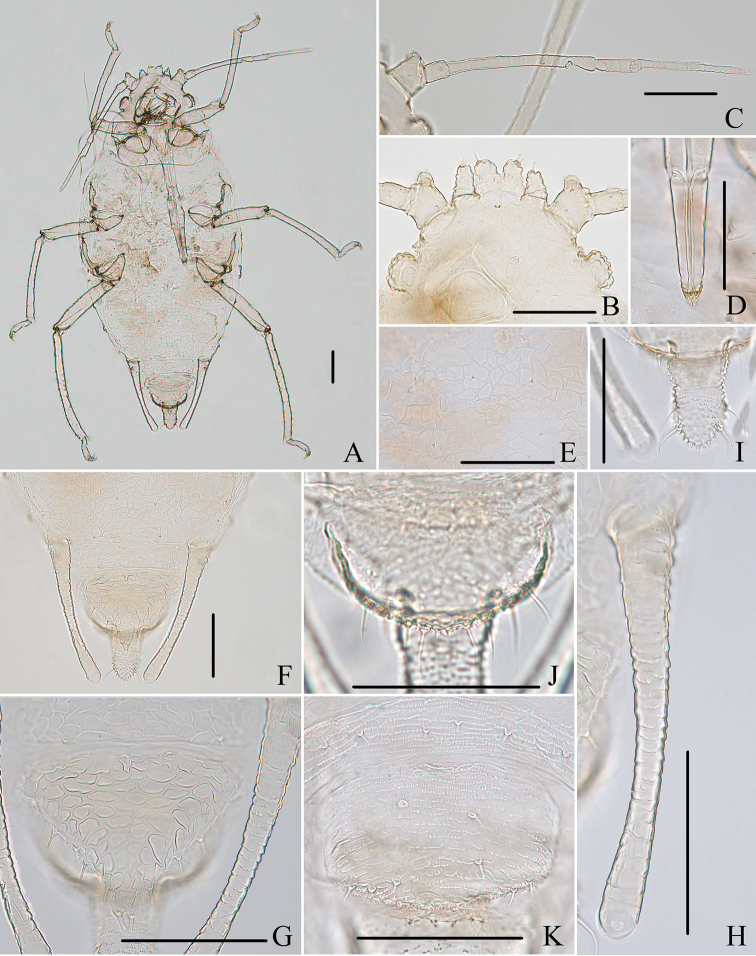
Aspidophorodon (Eoessigia) longirostre Qiao & Xu, sp. nov. Apterous viviparous female **A** dorsal view of body **B** dorsal view of head **C** antenna **D** ultimate rostral segment **E** oval and irregular polygonal sculptures of abdominal tergites **F** dorsal view of abdominal tergites V–VIII **G** spinal process of abdominal tergite VIII **H** siphunculus **I** cauda **J** anal plate **K** genital plate. Scale bars: 0.10 mm.

Figs 16 , 17 , Table 3

#### Specimens examined.

***Holotype***: apterous viviparous female, China: Sichuan (Baoxing City), 18.VIII.2003, No. 15089-1-2-1, on *Salix* sp., coll. K. Guo. ***Paratypes***: two apterous viviparous females (slides) and one apterous viviparous female (COI: OK668432), No. 15089-1-1, with the same collection data as holotype (NHMUK).

#### Diagnosis.

Dorsum of body covered with oval sculptures; median frontal tubercle well-developed, imbricated, with a strong depression at the middle into two cylinders; antennal tubercles each with a short finger-shaped and imbricated process at inner apex, lower than median frontal tubercle; rostrum reaching hind coxae, URS long wedge-shaped, long and thin; URS 3.28–3.42 × as long as its width, 1.56–1.92 × as long as 2HT; tergite VIII produced caudad into triangular spinal process reaching the middle of the cauda and constricted at apex and with distinctly oval sculptures.

#### Description.

Apterous viviparous females: body elongated oval (Fig. 17A).

**Mounted specimens.** Body pale in color (Fig. 17A). See Table 3 for General measurements.

***Head*.** Ocular tubercles small. Dorsum of head covered with oval sculptures, venter with wrinkles. Median frontal tubercle well-developed, imbricated, with a strong depression at middle separating it into two cylinders (Figs 16A, 17B), each cylinder with one pair of long and blunt setae at apex. Antennal tubercles undeveloped, each with a short finger-shaped and imbricated process at inner apex, the apex is blunt, as high as median frontal tubercle, each with a long and blunt seta at apex (Figs 16A, 17B). Dorsal setae of head short and capitate, with small setal tubercles. Head with one pair of dorsal setae between antennae, and two pairs of dorsal setae between compound eyes arranged transversely. Antennae 4-segmented, Ant. I distinctly projected into short cylindrical at inner apex (Figs 16B, 17C), 0.014–0.023 mm, with two short and blunt setae at apex; Ant. I–II smooth, with slight wrinkles, Ant. III–IV with imbrications (Figs 16B, 17C). Antennal setae short and blunt, Ant. I–IV with 3–4, 3–4, 3–4, 1–3 (base) +1 (PT) setae, respectively; apex of PT with two or three setae. Primary rhinaria unciliated. Rostrum reaching hind coxae, with apex pale brown; URS long wedge-shaped, long, and thin (Figs 16C, 17D), with three pairs of primary setae and two or three accessory setae.

***Thorax*.** Pronotum with oval and wavy sculptures; meso- and metanotum with oval sculptures on spinal area, pleura-marginal area with wavy and irregular polygonal sculptures. Thoracic setae sparse, short, blunt or capitate, with small setal tubercles; pronotum with two pairs of spinal setae, arranged anteriorly and posteriorly, one pair of pleural and one pair of marginal setae; meso- and metanotum each with one pair of spinal, one pair of pleural, and two pairs of marginal setae. Legs normal; coxae and femora smooth, distal parts of tibiae slightly imbricated. Setae on 2/3 distal part of femora and tibiae, short and blunt; hind tibiae with a row of short and blunt setae dorsally on middle. First tarsal chaetotaxy: 3, 3, 2. Second tarsal segments slightly imbricated.

***Abdomen*.** Abdominal tergites I–VII with oval and irregular polygonal sculptures (Fig. 17F, G); tergite VIII with distinctly oval sculptures, produced caudad into triangular spinal process reaching the middle of the cauda and constricted at apex (Figs 16D, 17G). Abdominal ventral plate with fine spinules arranged in rows. Dorsal setae of abdomen short, capitate or blunt, with small bases, ventral setae short and pointed. Abdominal tergites I–II each with one pair of spinal, pleural, and marginal setae; tergites III–VII each with one pair of spino-pleural and one pair of marginal setae; tergite VIII with 9–12 setae at margin. Spiracles reniform, open; spiracular plates slightly swollen. SIPH long spoon-shaped, incurved inward, broad at base, thin at middle, slightly swollen distally, with distinct imbrications, obliquely truncated at tip, without flange (Figs 16E, 17H). Cauda wide tongue-shaped, slightly constricted at the middle, with spinulose imbrications and four setae (Figs 16F, 17I). Anal plate semicircular, spinulose (Figs 16G, 17J), with 11–14 setae. Genital plate broadly round, with sparse spinules in transverse rows (Figs 16H, 17K), with two anterior setae and 4-6 setae along the posterior margin.

**Fourth instar apterous nymph.** As in apterous viviparous females except as follows: legs normal; femora scabrous at distal part, and tibia with spinulose imbrications distributed on 2/3 distal part. Setae on legs short and blunt; hind tibiae with long pointed setae dorsally and short blunt setae ventrally, and with a row of short and blunt setae dorsally on the middle.

#### Etymology.

The new species is named for its long URS, *longirostre* being the neuter form of the adjective.

#### Taxonomic notes.

The new species resembles *A.indicum* (David, Rajasingh & Narayanan) in median frontal tubercle protuberant; dorsum of head covered with distinctly oval and wavy sculptures; abdominal tergite VIII with a spinal tubercle; but differs from it as follows: median frontal tubercle well-developed, imbricated, with a strong depression at the middle separating it into two cylinders, a finger-shaped and imbricated process at inner apex of antennal tubercles (the latter: median frontal tubercle protuberant rectangular and slightly depressed at the middle, a short cylindrical process at inner apex of antennal tubercles); abdominal tergite VIII produced caudad into triangular process (the latter: abdominal tergite VIII with conical spinal process); dorsum of head covered with oval sculptures (the latter: dorsum of head with densely semicircular and wavy sculptures).

The new species resembles *A.furcatum* Qiao & Xu, sp. nov. in well-developed median frontal tubercle, with a strong depression at middle separating it into two cylinders; abdominal tergite VIII produced caudad into triangular spinal process; SIPH long spoon-shaped, curved inward; cauda wide, tongue-shaped, slightly constricted at the middle. The new species differs from *A.furcatum* as follows: median frontal tubercle protuberant, 0.025–0.046mm; a finger-shaped and blunt process at inner apex of antennal tubercles, 0.027-0.047mm, as high as median frontal tubercle (the latter: median frontal tubercle well-developed, 0.063–0.077mm; a long finger-shaped and pointed process at inner apex of antennal tubercles, 0.077–0.095mm, higher than median frontal tubercle); rostrum reaching hind coxae, URS 3.28–3.42 × as long as its width, 1.56–1.92 × as long as 2HT (the latter: rostrum reaching mid-coxae, URS 2.21–3.18 × as long as its width, 1.31–1.62 × as long as 2HT); abdominal tergite VIII with oval sculptures, constricted at apex (the latter: abdominal tergite VIII with distinctly irregular polygonal makings and marginal area with wavy sculptures, blunt at apex).

#### Host plant.

*Salix* sp.

#### Distribution.

China (Sichuan).

#### Biology.

This species colonizes the undersides of leaves of its host plant.

### ﻿ Aspidophorodon (Eoessigia) longituberculatum

Taxon classificationAnimaliaHemipteraAphididae

(Zhang, Zhong & Zhang, 1992)

1B2CAFCD-B7EA-54B7-B27F-7A9B71448D34

Figs 18 , 21B–E

Margituberculatus longituberculatum Zhang, Zhong & Zhang 1992: 382; Remaudière and Remaudière 1997: 117; Blackman and Eastop 2006: 1219.
Aspidophorodon (Aspidophorodon) cornuatum Qiao: Chen et al. 2015: 558. Syn. nov.
Aspidophorodon (Eoessigia) longituberculatum (Zhang, Zhong & Zhang): Chen et al. 2015: 570.

#### Specimens examined.

One alate viviparous female (Holotype), China: Yunnan (Lijiang City, Mt. Yulongxue, altitude 2900 m), 27.V.1980, No. 7165-1-1-1, on *Polygonum* sp., coll. T.S. Zhong and L.Y. Wang; Holotype and paratypes of *Aspidophorodoncornuatum* Qiao, 2015 syn. nov.: one apterous viviparous female, China: Tibet (Yadong County, 27.52°N, 88.97°E, altitude 2800 m), 15.VIII.2010, No. 25908-2-3-1, on *Salixcupularis*, coll. Y. Wang; five apterous viviparous females, with the same collection data as holotype. Other materials: one alate viviparous female, China: Tibet (Yadong County), 11.VII.2014, 32672-1-1, on *Salix* sp., coll. J. Chen and X.C. Zhu; two apterous viviparous females, China: Tibet (Motuo County), 16.IX.2020, No. 49262-1-1, on *Salix* sp., coll. Y. Xu.; one apterous viviparous female and one alate viviparous female (slide), one apterous viviparous female (COI: OK668444), one alate viviparous female (COI: OK668445), China: Tibet (Bomi County), 27.VI.2021, 51707-1-1, on *Salix* sp., coll. Y. Xu.

#### Comments.

**Figure 18.**
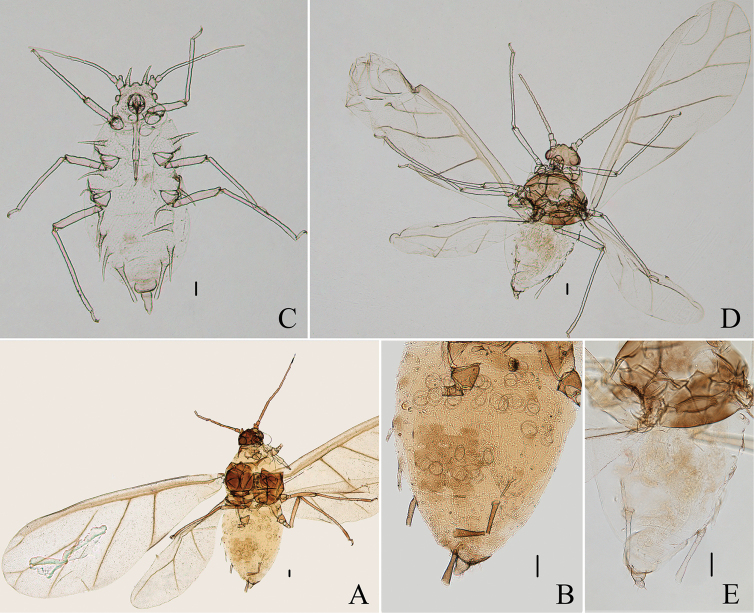
Aspidophorodon (Eoessigia) longituberculatum (Zhang, Zhong & Zhang, 1992). Alate viviparous female **A** dorsal view of body **B** dorsal view of abdomen. Apterous viviparous female **C** dorsal view of *Aspidophorodoncornutus* Qiao, 2015 syn. nov. Alate viviparous female **D** dorsal view of *Aspidophorodoncornutum* Qiao, 2015 syn. nov. **E** dorsal view of abdomen of *Aspidophorodoncornutum* Qiao, 2015 syn. nov. Scale bars: 0.10 mm.

The species was erected in genus *Margituberculatus* based on only one alate viviparous female (Zhang et al. 1992). Then the species was removed to the genus *Aspidophorodon* as *Aspidophorodonlongituberculatum* according to the characters of processes and siphunculi; meanwhile, *Aspidophorodoncornuatum* was described as a new species (Chen et al. 2015). At that time, there were no alate viviparous females of *Aspidophorodoncornuatum*, so it was difficult to compare with the two species. After several surveys in southwest China, apterous viviparous female (Fig. 18C) and alate viviparous female (Fig. 18D) of *Aspidophorodoncornuatum* were collected. The alate viviparous female of *Aspidophorodoncornuatum* is with marginal processes on abdominal tergites I–IV (Fig. 18E) which is the same as *Aspidophorodonlongituberculatum* (Fig. 18A, B). The molecular data of alate viviparous females of *Aspidophorodonlongituberculatum* and apterous viviparous females of *Aspidophorodoncornuatum* support they are the same species (Fig. 23). So, *Aspidophorodoncornuatum* Qiao, 2015 should be considered as junior synonym of *Aspidophorodonlongituberculatum* (Zhang, Zhong & Zhang, 1992).

*Aspidophoron* being neuter, the adjectival specific epithet is also neuter, so *longituberculatus* is revised as *longituberculatum*.

#### Host plant.

*Salixcupularis*.

#### Distribution.

China (Yunnan, Tibet).

#### Biology.

The species occurs on the undersides of leaves along the main vein of host plants (Fig. 21B–E).

### ﻿ Aspidophorodon (Eoessigia) sorbi

Taxon classificationAnimaliaHemipteraAphididae

(Chakrabarti & Maity, 1984)

24715A36-4F30-57D5-B9B7-78BDEBA6B8D0

Indotuberoaphis sorbi Chakrabarti & Maity 1984: 198; Blackman and Eastop 1994: 727; Remaudière and Remaudière 1997: 104.
Aspidophorodon (Eoessigia) sorbi (Chakrabarti & Maity): Stekolshchikov and Novgorodova 2010: 43.

#### Host plant.

*Sorbusfoliolosa*.

#### Distribution.

India.

#### Biology.

This species occurs on the undersides of young leaves of *Sorbusfoliolosa*. No ant-attendance was noticed (Chakrabarti and Maity 1984).

### ﻿ Aspidophorodon (Eoessigia) obtusirostre

Taxon classificationAnimaliaHemipteraAphididae

Qiao & Xu sp. nov.

91ABC474-086C-5811-98F1-4C2CEAC2056E

http://zoobank.org/47423FD8-010A-4191-B9CA-A17C67273CB9

**Figure 19.**
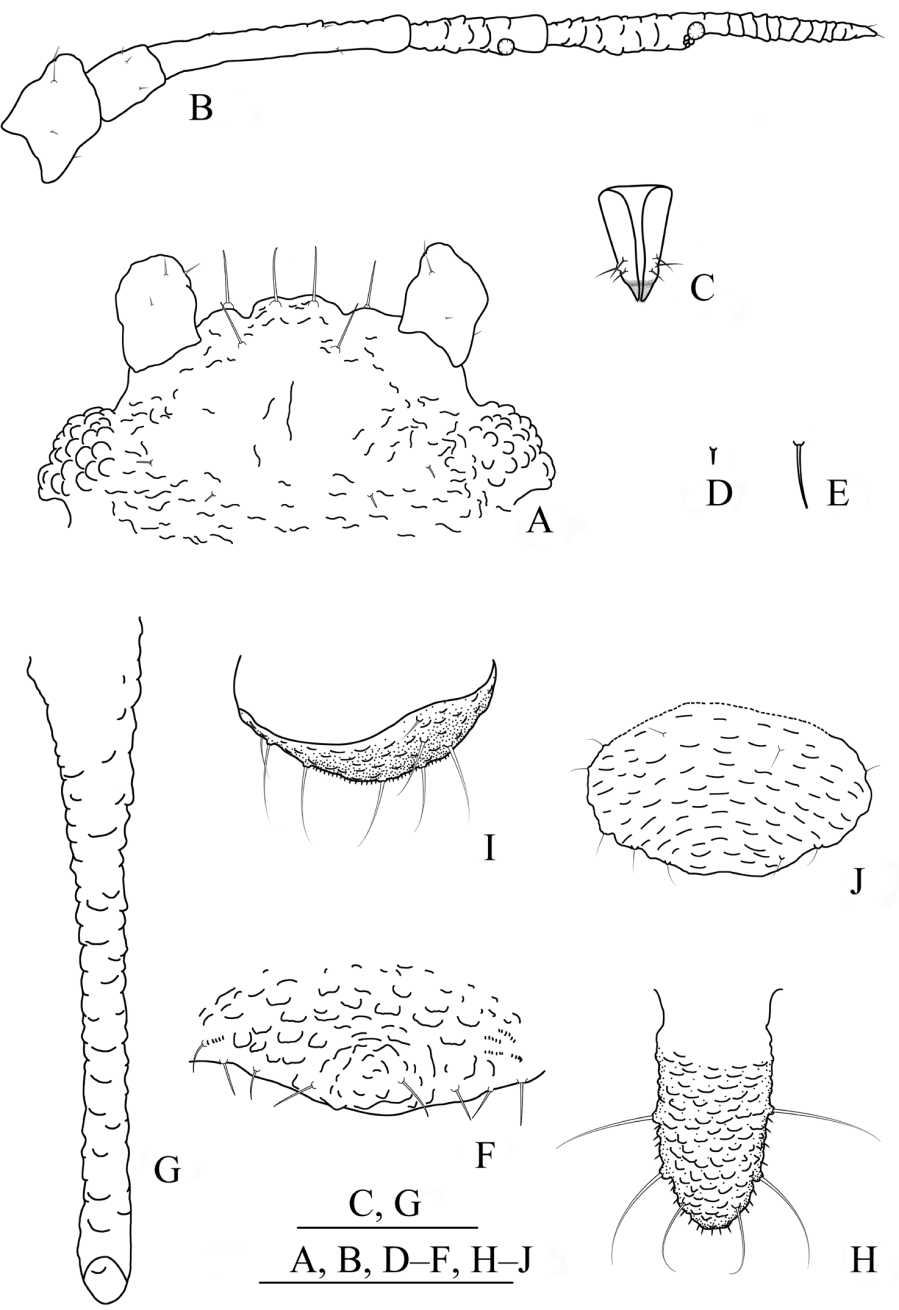
Aspidophorodon (Eoessigia) obtusirostre Qiao & Xu, sp. nov. Apterous viviparous female **A** dorsal view of head **B** antenna **C** ultimate rostral segment **D** marginal seta of abdominal tergite I **E** spinal seta of abdominal tergite VIII **F** spinal process of abdominal tergite VIII **G** siphunculus **H** cauda **I** anal plate **J** genital plate. Scale bars: 0.10 mm.

**Figure 20.**
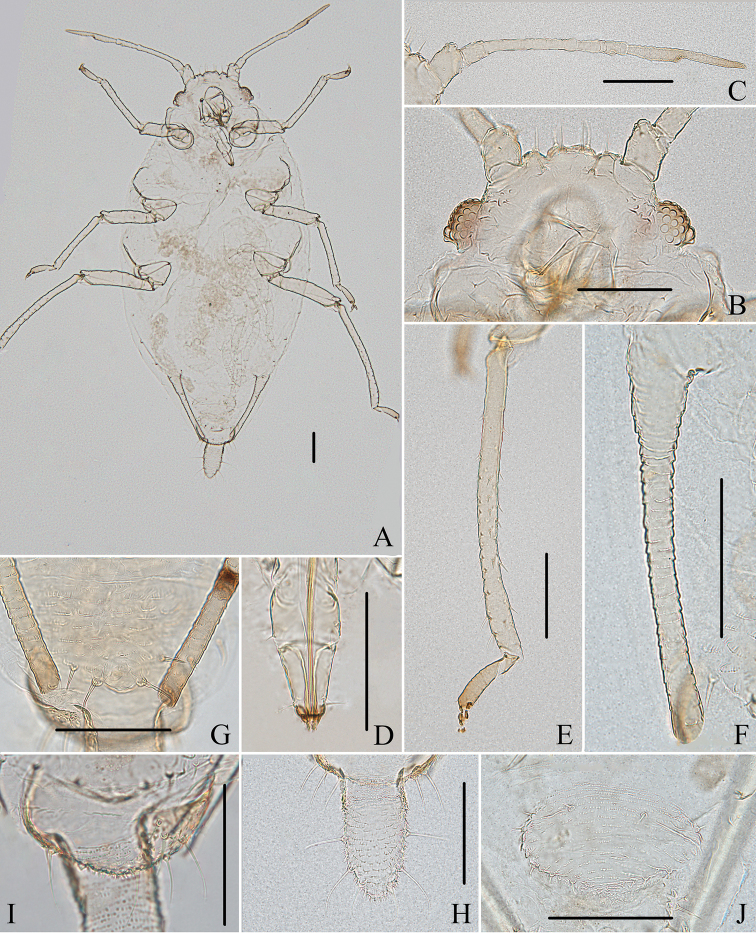
Aspidophorodon (Eoessigia) obtusirostre Qiao & Xu, sp. nov. Apterous viviparous female **A** dorsal view of body **B** dorsal view of head **C** antenna **D** ultimate rostral segment **E** hind tibia and tarsi **F** siphunculus **G** spinal process of abdominal tergite VIII **H** cauda **I** anal plate **J** genital plate. Scale bars: 0.10 mm.

Figs 19 , 20 , Table 3

#### Specimens examined.

***Holotype***: apterous viviparous female, China: Beijing (Mt. Dongling, 40.03°N, 115.42°E, altitude 2063m), 24.VIII.2015, No. 35918-1-1; on *Potentilla* sp., coll. H. Long; ***Paratypes***: five apterous viviparous females (slides) and one apterous viviparous female (COI: OK668433), 35918-1-2 with the same collection data as holotype; two apterous viviparous females, 35918-1-3, with the same collection data as holotype (NHMUK).

#### Diagnosis.

Median frontal tubercle protuberant, rectangular, with a shallow depression at middle; antennal tubercles each with a low process at inner apex, lower than median frontal tubercle; rostrum reaching mid-coxae, URS wedge-shaped, short and blunt, 1.27–1.94 × as long as its width, 0.70–0.84 × as long as 2HT; cauda long tongue-shaped with 6–11 setae, including two pairs of very long and pointed setae and 2–7 short and pointed setae.

#### Description.

Apterous viviparous females: body elongated oval (Fig. 20A), yellowish in life.

**Mounted specimens.** Body pale, PT, distal part of rostrum, tarsi, distal parts of SIPH and anal plate pale brown, other parts pale in color (Fig. 20A). See Table 3 for general measurements.

***Head*.** Ocular tubercles small. Dorsum of head covered with wavy sculptures (Figs 19A, 20B), those distinctly developed between compound eyes. Median frontal tubercle protuberant, rectangular, with a shallow depression at middle (Figs 19A, 20B), with one pair of thick and blunt setae on venter. Antennal tubercles undeveloped, each with a low process at inner apex, and lower than median frontal tubercle (Figs 19A, 20B), each process with a thick and blunt seta at apex, occasionally with two thick and blunt setae. Head with one pair of dorsal setae between antennae, thick and blunt, and two pairs of dorsal setae between compound eyes arranged transversely, short and blunt. Antennae 5-segmented, Ant. I slightly projected at inner apex, Ant. I–III smooth, Ant. IV–V slightly imbricated (Figs 19B, 20C). Antennal setae short and blunt, Ant. I–V with 4–5, 3–4, 1–2, 0–2, 0–2 (base) +0–1 (PT) setae, respectively; apex of PT with two or three setae. Primary rhinaria ciliated. Rostrum reaching mid-coxae; URS wedge-shaped, short and blunt (Figs 19C, 20D), with three pairs of primary setae, and without accessory setae.

***Thorax*.** Prothorax nota with wrinkles, those developed on spino-pleural area. Meso- and metanotum with wrinkles on marginal area, spino-pleural area smooth. Thoracic setae sparse, short and blunt, with small setal tubercles; pronotum with two pairs of spinal setae, arranged in anterior and posterior pairs, one pair of pleural and one pair of marginal setae; meso- and metanotum each with one pair of spinal, one pair of pleural setae, two pairs of marginal setae, respectively. Legs normal, smooth. Setae on 2/3 distal part of femora and tibiae, short and blunt; hind tibiae with a row of short and blunt setae dorsally on the middle (Fig. 20E). First tarsal chaetotaxy: 3, 2, 2. Second tarsal segments slightly imbricated.

***Abdomen*.** Abdominal tergites I–VII with wrinkles, those distinctly developed on marginal area; tergite VIII with scaly sculptures, swollen into conical spinal process, with 7–10 long, thick, and blunt setae at margin (Figs 19F, 20G). Venter of abdominal tergites III–VIII with fine spinules arranged in rows. Dorsal setae of abdominal tergites I–VI short, thick, and blunt (Fig. 19D), tergite VII long, thick, and blunt, occasionally short, thick, and blunt, tergite VIII long, thick, and blunt with distinct setal tubercles (Fig. 19E); ventral setae short and pointed. Abdominal tergites I and II each with one pair of spinal, pleural, and marginal setae; tergites III–VII each with one pair of spinal and marginal setae. Spiracles reniform, open or closed; spiracular plates slightly swollen. SIPH long spoon-shaped, broad at base, thin at the middle, swollen distally, with developed imbrications, obliquely truncated at tip, without flange (Figs 19G, 20F). Cauda long tongue-shaped, with spinulose imbrications, slightly constricted at base and weakly pointed at apex (Figs 19H, 20H); with 6–11 setae, including two pairs of very long and pointed setae, 0.055–0.061mm and 2–7 short and pointed setae, 0.027–0.041mm. Anal plate semicircular, spinulose (Figs 19I, 20I), with 8–13 setae. Genital plate transversely oval, with sparse spinules in transverse stripes (Figs 19J, 20J), with 4 or 5 anterior setae and 5–7 setae along the posterior margin.

#### Etymology.

The new species is named for its short and blunt URS. The Latin word *obtus* means blunt, and *rostre* for rostrum, *obtusirostre* being the neuter form of the adjective.

#### Taxonomic notes.

The new species resembles *A.indicum* (David, Rajasingh & Narayanan) in median frontal tubercle protuberant, rectangular; dorsal setae of head between antennal tubercles thick and blunt; abdominal tergite VIII with conical spinal process; SIPH long spoon-shaped; but differs from it as follows: dorsum of head covered with wavy sculptures, those distinctly developed between compound eyes, thoracic nota and abdominal tergites I–VII with wavy sculptures (the latter: dorsum of head with densely semicircular and wavy sculptures, thoracic nota, and abdominal tergites I–VII with semicircular and wavy sculptures); antennae 5-segmented, 0.30–0.36 × as long as body length (the latter: antennae 6-segmented, 0.38–0.52 × as long as body length); URS short and blunt, 1.27–1.94 × as long as the basal width, 0.70–0.84 × as long as 2HT (the latter: URS long wedge-shaped, 2.06–2.54 × as long as the basal width, 0.89–1.10 × as long as 2HT).

#### Host plant.

*Potentilla* sp.

#### Distribution.

China (Beijing).

#### Biology.

The species colonizes the undersides of leaves of its host plant and with ant-attendance.

### ﻿ Aspidophorodon (Eoessigia) vera

Taxon classificationAnimaliaHemipteraAphididae

Stekolshchikov & Novgorodova, 2010

E5757309-9030-57D5-ACEA-39B1DE40B2F0

Aspidophorodon (Eoessigia) vera Stekolshchikov & Novgorodova, 2010: 39.

#### Host plant.

*Potentillafruticosa*.

#### Distribution.

Russia (the Altai Republic).

**Figure 21.**
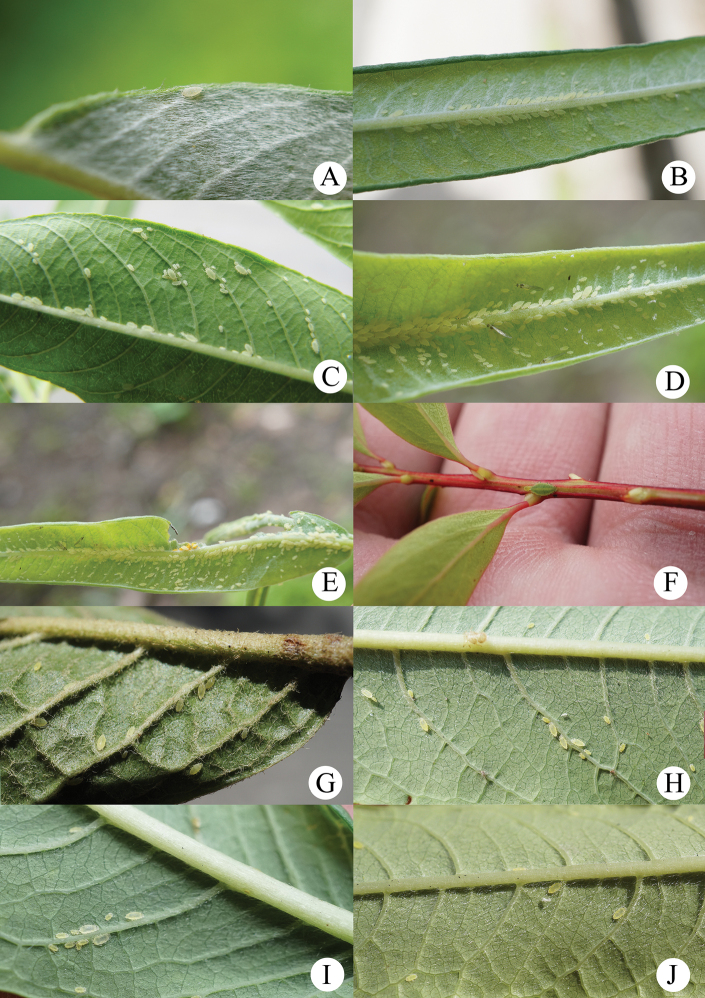
The ecological photos of *Aspidophorodon* in the field **A** an aptera of *Aspidophorodoncapitatum* Qiao & Xu, sp. nov. on underside of leaf **B, C** the apterae and nymphs of *Aspidophorodonlongituberculatum* (Zhang, Zhong & Zhang) on main veins and part lateral veins of underside of leaf **D, E** the apterae, alatae and nymphs of *Aspidophorodonlongituberculatum* (Zhang, Zhong & Zhang) on main veins and part lateral veins of underside of leaves **F** an aptera and a nymph of *Aspidophorodonharvense* Verma on a twig **G** the apterae of *Aspidophorodonobtusum* Qiao on underside of leaf **H–J** the apterae and nymphs of *Aspidophorodonsalicis* Miyazaki on underside of leaf.

#### Biology.

The species feeds along the margins on the underside of leaves of its host plant (Stekolshchikov and Novgorodova 2010).

**Figure 22.**
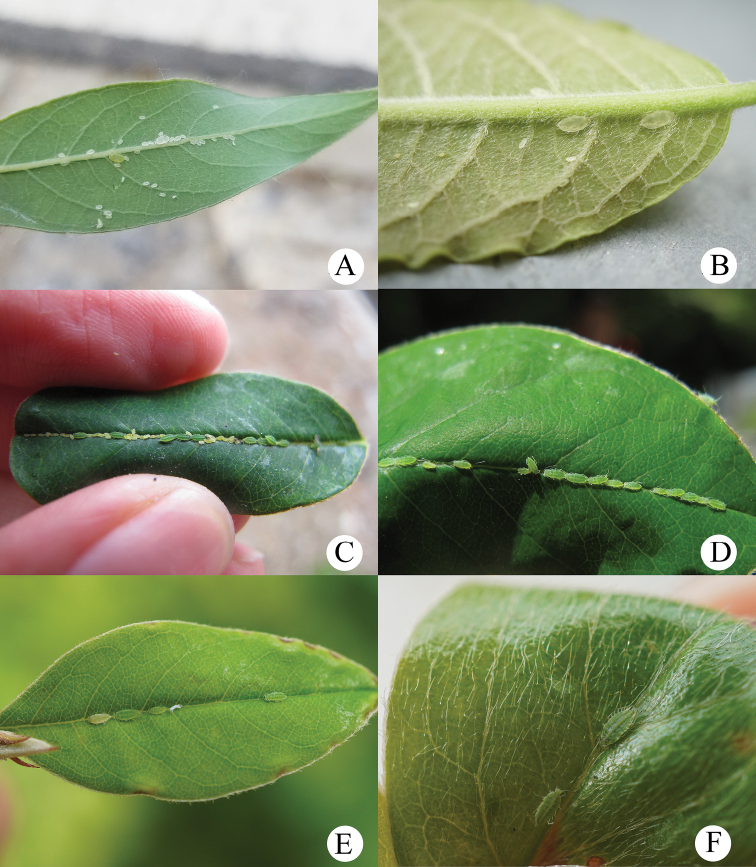
The ecological photos of *Aspidophorodon* in the field **A, B** the apterae and nymphs of *Aspidophorodonfurcatum* Qiao & Xu, sp. nov. on undersides of leaves **C–E** the apterae and nymphs of *Aspidophorodonindicum* (David, Rajasingh & Narayanan) on main veins of upperside of leaves **F** the fundatrices of *Aspidophorodonindicum* (David, Rajasingh & Narayanan) on main vein of upperside of leaf.

##### ﻿DNA barcoding

**Figure 23.**
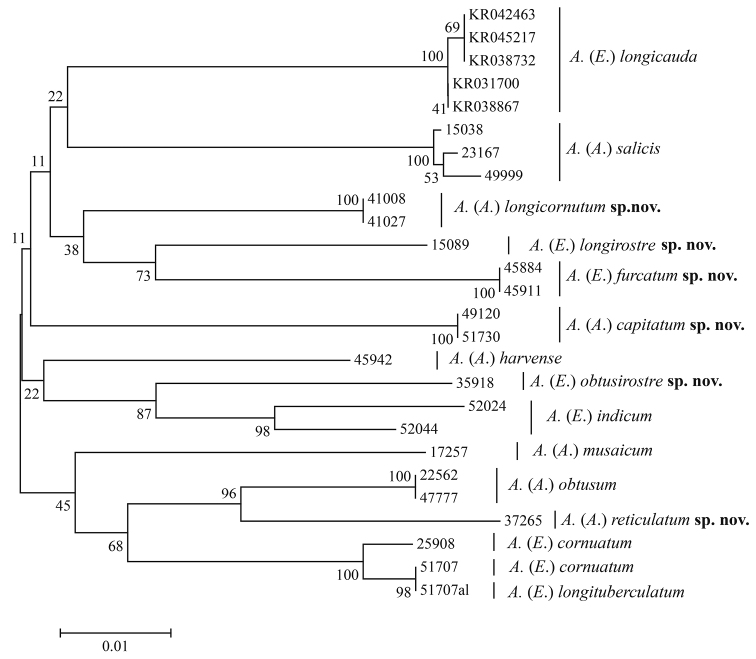
Neighbour-joining tree for *Aspidophorodon* samples based on COI sequences.

The final alignments of COI sequences consisted of 658 nucleotides, including 119 parsimony-informative sites. Pairwise sequence divergences of the gene among the *Aspidophorodon* species are presented in Table 4. The interspecific genetic distances of new species and known species averaged 6.98% (range: 3.93%-8.97%) for COI closely corresponding to the divergence of *Aspidophorodon* taxa base on four species (mean: 6.88%; range: 5.29%-7.68%) (Chen et al. 2015). The validity of species was well-supported on NJ tree (>95% bootstrap) (Fig. 23). At the same time, *Aspidophorodoncornuatum* and *Aspidophorodonlongituberculatum* formed a clade (Fig. 23) and the genetic distance between the two species is 0.00%–0.46%, so the result proved *A.cornuatum* was a junior synonym of *A.longituberculatum*. However, the subgenera were not monophyletic groups on NJ tree, and this needs more evidence and more samples to prove. In this study, we also followed the traditional taxonomic system to divide two subgenera in *Aspidophorodon*. According to the distinct morphological characteristics in description and interspecific genetic distances between species, the six new species were supported.

**Table 4.** Kimura’s two-parameter genetic distances among *Aspidophorodon* species samples based on COI.

	1	2	3	4	5	6	7	8	9	10	11	12	13
1. *A.capitatum* sp. nov.													
2. *A.cornuatum* syn. nov.	0.082												
3. *A.furcatum* sp. nov.	0.079	0.085											
4. *A.harvense*	0.069	0.058	0.072										
5. *A.indicum*	0.079	0.065	0.076	0.070									
6. *A.longicauda*	0.075	0.079	0.078	0.075	0.073								
7. *A.longicornutum* sp. nov.	0.069	0.070	0.064	0.054	0.072	0.066							
8. *A.longirostre* sp. nov.	0.077	0.075	0.056	0.062	0.070	0.070	0.056						
9. *A.longituberculatum*	0.080	0.005	0.085	0.057	0.066	0.080	0.069	0.076					
10. *A.musaicum*	0.074	0.060	0.077	0.065	0.081	0.075	0.072	0.076	0.061				
11. *A.obtusirostre* sp. nov.	0.076	0.067	0.079	0.057	0.052	0.078	0.070	0.074	0.067	0.084			
12. *A.obtusum*	0.074	0.050	0.064	0.062	0.074	0.078	0.069	0.081	0.047	0.065	0.081		
13. *A.reticulatum* sp. nov.	0.079	0.062	0.086	0.072	0.080	0.077	0.071	0.090	0.061	0.072	0.086	0.039	
14. *A.salicis*	0.079	0.074	0.088	0.073	0.072	0.071	0.063	0.064	0.073	0.068	0.086	0.075	0.085

## ﻿Discussion

The species in *Aspidophorodon* were identified by stable characters: the shape and variability of processes on the frons, the form of markings on the dorsum, the shape of the ultimate rostral segment, the sculptures on the siphunculi, and the shape of the cauda. Some species in the genus have developed spinal and marginal processes on the abdominal tergites. The presence or absence of the spinal and marginal processes on abdominal tergites are inconsistent within a species, but the shape of processes is consistent. *Aspidophorodonsalicis* has short conical marginal processes on abdominal tergites I–IV in the fundatrix, whereas the apterous viviparous female and the alate viviparous female are without such processes. The populations of *Aspidophorodonobtusum* feeding on *Salix* sp. have cylindrical marginal processes on abdominal tergites I–IV, while the ones feeding on *Cotoneaster* sp. have no marginal processes. *Aspidophorodonindicum* has long conical marginal processes on abdominal tergites I–IV and spinal processes on abdominal tergite VIII in the fundatrix, whereas the apterous viviparous female and the alate viviparous female have no marginal processes and shorter spinal processes on abdominal tergite VIII. Hence, the processes tend to reduce in size during the life of the colony. The median frontal tubercle, processes on antennal tubercles, and sculptures of the body are relatively stable to enable identification of the species.

## Supplementary Material

XML Treatment for Aspidophorodon

XML Treatment for Aspidophorodon

XML Treatment for Aspidophorodon capitatum

XML Treatment for Aspidophorodon harvense

XML Treatment for Aspidophorodon longicornutum

XML Treatment for Aspidophorodon musaicum

XML Treatment for Aspidophorodon obtusum

XML Treatment for Aspidophorodon reticulatum

XML Treatment for Aspidophorodon salicis

XML Treatment for Eoessigia

XML Treatment for Aspidophorodon (Eoessigia) furcatum

XML Treatment for Aspidophorodon (Eoessigia) indicum

XML Treatment for Aspidophorodon (Eoessigia) longicauda

XML Treatment for Aspidophorodon (Eoessigia) longirostre

XML Treatment for Aspidophorodon (Eoessigia) longituberculatum

XML Treatment for Aspidophorodon (Eoessigia) sorbi

XML Treatment for Aspidophorodon (Eoessigia) obtusirostre

XML Treatment for Aspidophorodon (Eoessigia) vera
